# Genetic loci responsible for atrioventricular block in children

**DOI:** 10.3389/fcell.2026.1845520

**Published:** 2026-09-11

**Authors:** Zhenhui Pan, Wanling Zhao, Fuqiang Liu, Yifei Li

**Affiliations:** Department of Pediatrics, Key Laboratory of Birth Defects and Related Diseases of Women and Children of MOE, West China Second University Hospital, Sichuan University, Chengdu, Sichuan, China

**Keywords:** arrhythmia, AVB, children, genetic disorders, review

## Abstract

Atrioventricular block (AVB) represents a prevalent form of bradyarrhythmia in the pediatric population, broadly classified into congenital and acquired subtypes. Historically, the underlying mechanisms of AVB were predominantly attributed to structural cardiac anomalies, maternal autoantibody-mediated inflammatory processes, and pharmacological agents; however, the contribution of genetic factors remained largely underexplored. With the advent and progressive refinement of genetic sequencing technologies, a growing repertoire of arrhythmia-associated genes has been systematically identified. In this review, we comprehensively examine the genetic determinants implicated in pediatric AVB, encompassing genes encoding cardiac ion channels, including SCN5A, KCNQ1/KCNJ2 (KCN family), KCNH2 (HERG), TRPM4, and HCN4, as well as genes governing cardiac structural integrity (GJA5/GJA1, ZO-1, NKX2-5, TBX3/TBX5, HAND1/HAND2, and ID family members), metabolic regulation (PRKAG2, PPARA, and LAMP2), and immune-mediated pathways (TRIM21/TROVE2 and HLA class II loci). The genetic architecture underlying pediatric AVB demonstrates considerable complexity, characterized by notable genotypic heterogeneity and “multi-phenotypic” expressivity, wherein distinct mutations within a single gene may manifest across a spectrum of clinical phenotypes. Collectively, these findings establish a molecular framework for elucidating the pathogenesis of AVB, while simultaneously offering valuable insights to inform clinical diagnosis, facilitate precision-based intervention strategies, and guide the development of targeted therapeutic modalities.

## Introduction

Pediatric heart diseases are broadly categorized into two major groups: structural heart disease and cardiac arrhythmias. The former encompasses conditions such as atrial septal defect, ventricular septal defect, and tetralogy of Fallot, while the latter includes sick sinus syndrome, atrioventricular block (AVB), paroxysmal supraventricular tachycardia, and premature contractions, among others (Li et al., 2023). Given the heterogeneity of these conditions and their distinct pathophysiological mechanisms, accurate classification remains fundamental to both clinical management and mechanistic investigation.

Among the spectrum of bradyarrhythmias encountered in the pediatric population, AVB represents one of the most clinically significant entities. It is broadly classified into congenital and acquired subtypes and, based on electrocardiographic characteristics, further stratified into first-, second-, and third-degree AVB. The pathophysiological hallmark of AVB is impaired atrioventricular conduction, which may result in a substantially reduced ventricular rate and consequent systemic hypoperfusion. Clinically, affected individuals may present with dizziness, fatigue, diminished exercise tolerance, and syncope; in severe cases, the condition may precipitate sudden cardiac death. In the pediatric context specifically, clinical manifestations may additionally include feeding difficulties, growth retardation, and dyspnea. Presentations that are particularly detrimental during critical developmental periods. Collectively, these sequelae impose a considerable burden on patients’ quality of life and place significant psychological and economic strain on affected families. Accordingly, early screening, timely preventive intervention, and comprehensive diagnostic and therapeutic strategies are of paramount clinical importance.

Prior to the widespread adoption of precision medicine, the pathogenesis of AVB was predominantly attributed to structural cardiac anomalies, maternal autoantibody-mediated immune-inflammatory responses, pharmacological agents, and idiopathic factors (Abu Rmilah et al., 2020; Uchino et al., 2020; Bello et al., 2009), with little attention directed toward deeper molecular mechanisms. In recent years, however, the rapid advancement of molecular biology techniques has facilitated the identification of an expanding number of genes implicated in the pathogenesis of pediatric AVB. The genetic basis of this condition was first suggested in 1995, when a chromosomal locus (19q13.2–13.3) was linked to progressive cardiac conduction disease (PCCD); nonetheless, the specific causative genes remained uncharacterized at that time. A pivotal breakthrough occurred in 1999, when SCN5A, encoding the cardiac voltage-gated sodium channel Nav1.5, which was identified as the first susceptibility gene associated with PCCD (Brink et al., 1995; Ripoll-Vera et al., 2015). Subsequently, the application of next-generation sequencing (NGS) technologies has substantially accelerated gene discovery in this field, revealing a diverse array of PCCD-associated genes encoding cardiac ion channels, regulatory proteins, protein kinases, structural proteins, and transcription factors.

**FIGURE 1:**
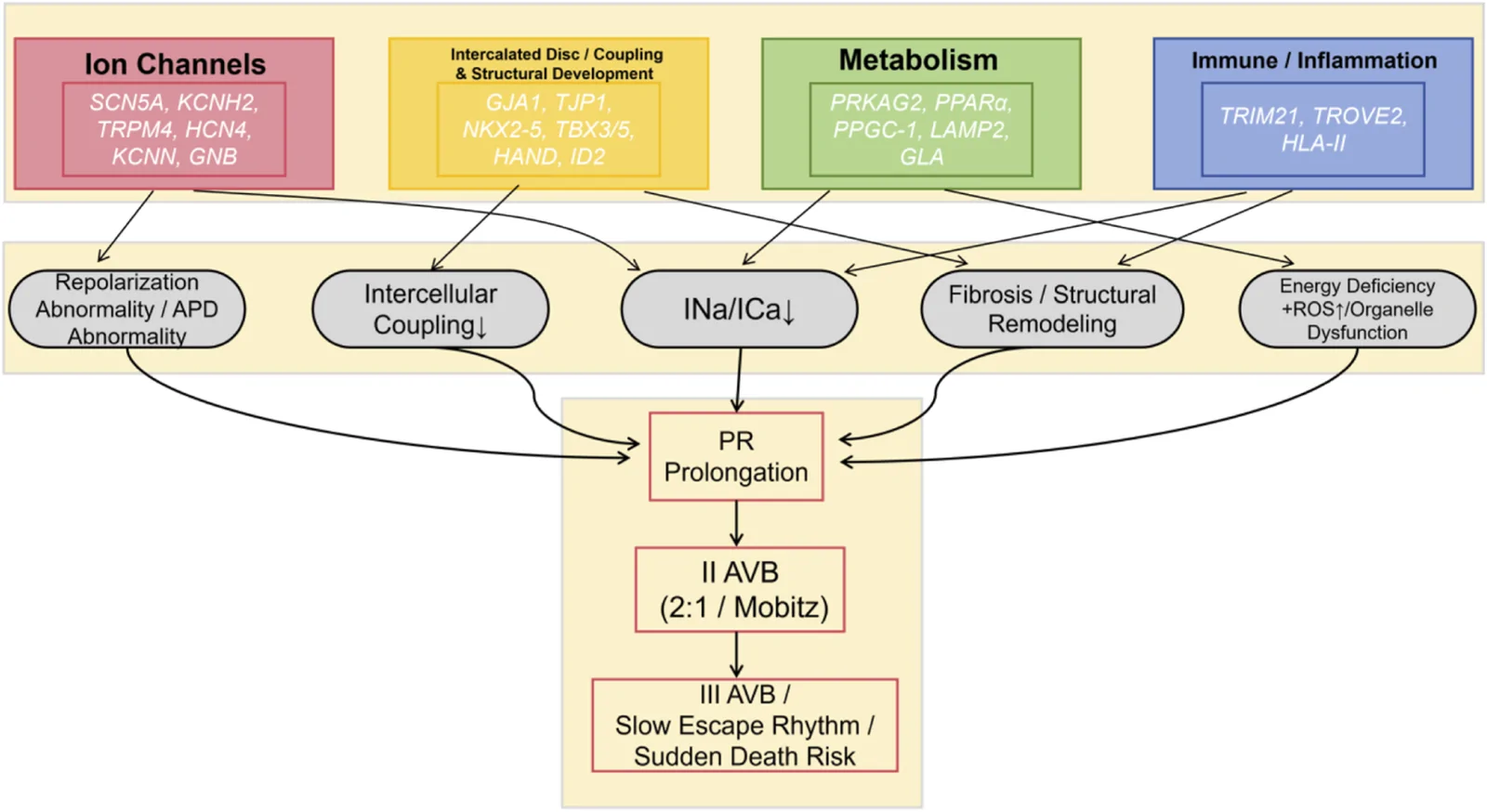
Overview diagram of Atrioventricular block mechanism. Abnormalities in ion channels (SCN5A, KCNH2), intercalated disc and structural development (GJA1, TJP1, NKX2-5), myocardial energy metabolism (PRKAG2, PPARα), and immune-inflammatory (TRIM21, TROVE2, HLA-II) responses collectively trigger electrophysiological disorders, impaired intercellular electrical coupling, reduced ionic currents, myocardial fibrosis and structural remodeling, as well as oxidative stress and energy metabolism dysfunction. These diverse pathways converge to prolong PR interval, progressing from second-degree AVB (Mobitz/2:1 block) to complete third-degree AVB, with associated risks of slow escape rhythm and sudden cardiac death.

In light of this rapidly evolving landscape, the present review systematically examines the known genetic determinants associated with AVB in children, delineates the underlying pathogenic mechanisms, and discusses their translational implications for clinical diagnosis and therapeutic decision-making (Figure 1).

## Cardiac ion channels

**FIGURE 2:**
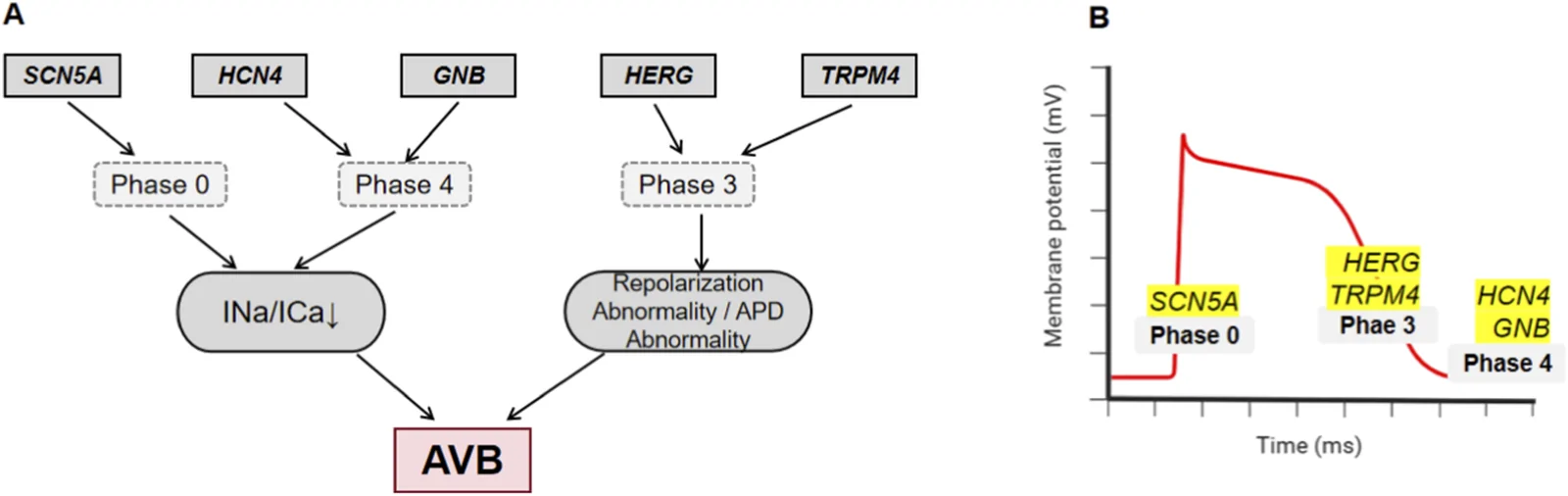
The mechanisms of ion channels dysfunction inducing AVB. **(A)**. Schematic diagram illustrating key ion channel genes involved in different phases of the cardiomyocyte action potential. SCN5A encodes the cardiac sodium channel responsible for Phase 0 depolarization. HCN4,and GNB contribute to Phase four diastolic depolarization and pacemaker activity. HERG and TRPM4 regulate Phase three repolarization. Pathogenic variants in these genes disrupt action potential kinetics, impair impulse generation and propagation, and collectively lead to AVB. **(B)**. The genes associated with AVB participating in different phases during action potential in cardiomyocyte.

The excitation and contraction of cardiomyocytes are governed by the coordinated activity of multiple ion channel systems, each fulfilling a distinct electrophysiological role across successive phases of the cardiac action potential. Specifically, rapid depolarization (Phase 0) is primarily mediated by voltage-gated fast sodium channels, while the plateau phase (Phase two) is sustained by L-type voltage-dependent calcium channels. Subsequent repolarization (Phase three) is driven by slowly activating delayed rectifier potassium channels, and the resting phase (Phase four) in automaticity-competent cells is characterized by the hyperpolarization-activated “funny” current, the inward rectifier potassium current, and T-type calcium channel currents, which collectively underpin spontaneous diastolic depolarization and pacemaker automaticity. Disruption of any of these channel systems through pathogenic genetic variants may critically impair impulse generation or conduction, thereby predisposing to AVB. In the following section, we systematically review the key genes encoding or regulating cardiac ion channels that have been implicated in the pathogenesis of AVB, with particular emphasis on their molecular mechanisms and associated clinical phenotypes (Figure 2).

### Sodium channel gene SCN5A

SCN5A is abundantly expressed in ventricular myocytes, atrial myocytes, and the cardiac conduction system (van den Boogaard et al., 2014), where its encoded protein, the cardiac voltage-gated sodium channel Nav1.5 — serves as the principal mediator of rapid membrane depolarization during Phase 0 of the cardiac action potential, functioning as the electrophysiological “igniter” of myocardial excitation. Pathogenic variants in the α-subunit of the sodium channel encoded by SCN5A have been associated with a broad spectrum of cardiac conduction abnormalities (Walsh et al., 2025; Qi et al., 2024; Villarreal-Molina et al., 2021). Loss-of-function mutations in this gene result in a reduction of the fast-inward sodium current (I_*Na*_) and accelerated channel deactivation, collectively diminishing Na^+^ influx during cardiomyocyte depolarization. As a consequence, both the upstroke velocity (dV/dt_*max*_) and peak amplitude of Phase 0 depolarization are attenuated, slowing conduction velocity throughout the cardiac conduction system and predisposing to varying degrees of AVB (Remme, 2023).

A well-characterized clinical manifestation of SCN5A loss-of-function is Lènegre disease (also known as progressive cardiac conduction disease, PCCD), an autosomal dominant disorder defined by progressive fibrotic degeneration of the conduction system (Zaytseva et al., 2024). Clinically, the disease typically follows a stereotyped progression: right bundle branch block (RBBB) emerges as the initial electrocardiographic manifestation, followed by gradual deterioration of conduction throughout the His–Purkinje system, ultimately culminating in complete atrioventricular block (Asatryan et al., 2019). To date, no disease-modifying therapy capable of halting this progressive fibrotic process has been established, and management remains largely symptomatic and stage-dependent. In the early stages, isolated RBBB or its combination with left anterior or posterior fascicular block generally does not produce significant hemodynamic compromise and, therefore, does not necessitate active intervention. Nevertheless, particular vigilance is warranted regarding the concurrent use of antiarrhythmic agents with inhibitory effects on conduction, such as sodium channel blockers, as these may paradoxically exacerbate conduction impairment in susceptible individuals; when such agents are deemed clinically necessary, initiation at a cautiously reduced dose is strongly advisable. In cases demonstrating rapid disease progression, glucocorticoid therapy may be considered as a temporizing measure, given its dual capacity to suppress fibrotic tissue proliferation and upregulate sodium channel opening, thereby transiently improving conduction (Remme, 2013). As the disease advances to high-degree or complete (third-degree) AVB, the anatomical site of block descends to a more distal level within the His–Purkinje system. Consequently, the ventricular escape pacemaker originates at an increasingly remote site from the block, exhibiting lower intrinsic automaticity and diminished chronotropic responsiveness, rendering the patient hemodynamically vulnerable. In patients who experience recurrent syncope or are at risk of sudden cardiac death attributable to severe ventricular arrhythmias secondary to profound bradycardia, implantation of a permanent pacemaker or an implantable cardioverter-defibrillator (ICD) should be considered as a definitive therapeutic strategy (Nordenswan et al., 2022; Surget et al., 2025; Proost et al., 2023).

Beyond Lènegre disease, SCN5A represents a pleiotropic arrhythmia susceptibility gene, with allelic variants also implicated in Brugada syndrome and congenital long QT syndrome type 3 (LQTS3), underscoring the broad phenotypic spectrum attributable to mutations within a single gene locus (Asatryan et al., 2019). Additionally, SCN10A, encoding the Nav1.8 sodium channel isoform—has been reported to influence PR interval duration through attenuation of cardiac action potential conduction velocity (van den Boogaard et al., 2014). Mechanistically, however, the predominant effect of SCN10 A appears to be exerted indirectly via transcriptional regulation of SCN5A expression in cardiac tissue, with the intrinsic electrophysiological contribution of its own gene product considered comparatively minor (van den Boogaard et al., 2014; Li et al., 2025).

### Voltage-gated potassium ion channel gene HERG

The *HERG* gene (*KCNH2*) is predominantly expressed in ventricular myocytes, with additional expression documented in atrial myocytes and throughout the cardiac conduction system (CCS). Its encoded protein constitutes the pore-forming α-subunit of a voltage-gated potassium channel responsible for generating the rapid component of the delayed rectifier potassium current (I_*Kr*​_), a current that plays an indispensable role in myocardial repolarization by facilitating the termination of the cardiomyocyte action potential and maintaining stable cardiac rhythm. Pathogenic variants in *KCNH2* that result in loss of I_*Kr*​_ function impair timely repolarization, thereby prolonging the action potential duration (APD) and the corrected QT interval (QTc) on surface electrocardiography.

Emerging evidence has established a significant association between *KCNH2* mutations and atrioventricular conduction disturbances in neonatal long QT syndrome (LQTS2), particularly in cases manifesting with 2:1 atrioventricular block (Moore et al., 2020; Shin et al., 2021). The underlying pathophysiology involves a self-reinforcing cycle of electrophysiological deterioration: severe AVB-induced bradycardia further prolongs the QT interval by extending the cardiac cycle length, which in turn amplifies APD prolongation. The resultant excessive prolongation of repolarization promotes enhanced L-type calcium channel (I_*Ca*,*L*_​) reactivation and the generation of early afterdepolarizations (EADs), both of which serve as triggering mechanisms for torsades de pointes (TdP) — a potentially fatal polymorphic ventricular tachyarrhythmia. This phenotypic constellation carries a particularly grave prognosis in the neonatal period, with reported infantile mortality rates exceeding 50%, underscoring the critical importance of early recognition and prompt therapeutic intervention.

For neonates and infants presenting with concomitant QT prolongation and 2:1 AVB, timely diagnosis and the institution of optimal management represent the most decisive determinants of clinical outcome. Pharmacological therapy constitutes the cornerstone of initial management, with non-selective β-adrenergic receptor blockers—such as propranolol—preferred over selective agents given their broader suppression of adrenergically triggered arrhythmic events; adjunctive therapy with mexiletine, a late sodium current (I_*Na*,*Late*_) inhibitor, may further abbreviate APD and attenuate the risk of EAD-mediated TdP (Shin et al., 2021; Milner et al., 2024). In cases refractory to pharmacological management or in patients with high-degree AVB compromising hemodynamic stability, permanent pacemaker implantation should be considered to maintain an adequate heart rate and mitigate bradycardia-dependent QT prolongation. Furthermore, Left Cardiac Sympathetic Denervation (LCSD) represents a viable adjunctive or alternative strategy in selected cases, particularly when β-blocker therapy is poorly tolerated or insufficient, by reducing the sympathetic adrenergic drive that potentiates arrhythmic vulnerability (Shin et al., 2021).

### Calcium ion activates the channel gene TRPM4

TRPM4 (Transient Receptor Potential Melastatin 4) is predominantly expressed in atrial and ventricular myocytes as well as Purkinje fibers (Hu et al., 2023), where it encodes a calcium-activated, non-selective monovalent cation channel that preferentially conducts Na^+^ ions across the sarcolemmal membrane. Under physiological conditions, TRPM4 channel activity contributes to the fine regulation of membrane potential by modulating the balance between inward sodium and outward potassium currents during the cardiac action potential. Gain-of-function (GOF) mutations in TRPM4 pathologically augment the inward Na^+^ current, thereby counteracting potassium efflux and impeding timely cellular repolarization, ultimately resulting in abnormal prolongation of the APD (Hu et al., 2023; Liu et al., 2010). Beyond direct electrophysiological perturbation, recent experimental evidence has further demonstrated that certain TRPM4 mutations may compromise protein stability and alter the half-life of the TRPM4 protein itself, leading to either upregulation or downregulation of channel expression at the sarcolemmal membrane (Bianchi et al., 2018). Such dysregulation of TRPM4 protein homeostasis consequently disrupts the orderly propagation of electrical impulses through the cardiac conduction system, representing an additional, and mechanistically distinct, pathway through which TRPM4 variants may precipitate conduction abnormalities.

The clinical relevance of TRPM4 dysfunction has been substantiated by numerous studies demonstrating a significant association between pathogenic TRPM4 variants and familial AVB (Dong et al., 2021; Palladino et al., 2022; Auricchio et al., 2023). Furthermore, a subset of Brugada syndrome patients has also been found to harbor TRPM4 mutations (Asatryan et al., 2019), highlighting the pleiotropic arrhythmogenic potential of this gene and its capacity to underlie phenotypically diverse cardiac conduction disorders. Collectively, these findings position TRPM4 as a critical determinant of cardiac electrophysiological integrity, and its dysregulation, whether through GOF electrophysiological effects or altered protein expression, converges on a common pathological outcome of impaired atrioventricular conduction.

Given the established pathophysiological significance of TRPM4 in cardiac electrical conduction and its association with a spectrum of arrhythmic disorders, the TRPM4 channel has emerged as a compelling therapeutic target for the development of novel antiarrhythmic strategies. In this context, sulfonylurea receptors (SURs), a class of regulatory subunits structurally and functionally coupled to ATP-sensitive potassium (K_*ATP*_) channels, warrant particular consideration (Amarouch and El Hilaly, 2020). Activation of SURs suppresses K_*ATP*_ channel opening, thereby reducing potassium efflux and elevating cardiomyocyte excitability. Notably, the functional and structural interplay between TRPM4 channels and SUR-associated K_*ATP*_ channel complexes suggests the existence of a modulatory relationship that may be pharmacologically exploitable. This mechanistic intersection offers a conceptual foundation for the rational design of targeted therapeutic agents directed at TRPM4 mutation-related cardiac diseases, potentially enabling more precise modulation of ion channel activity than is achievable with currently available antiarrhythmic pharmacotherapy.

### Cyclic nucleotide gated channel gene HCN4

HCN4 (Hyperpolarization-activated Cyclic Nucleotide-gated channel 4) is predominantly expressed in the sinoatrial (SA) node and atrioventricular (AV) node, the two principal pacemaking and conduction structures of the cardiac conduction system (Hennis et al., 2022). The protein encoded by HCN4 constitutes the primary molecular correlate of the hyperpolarization-activated “funny” current (I_*f*_), which plays a fundamental role in the spontaneous diastolic depolarization of Phase four in autorhythmic cardiomyocytes. Mechanistically, the HCN4 channel is a non-selective cation channel that permits the concurrent inward conductance of both Na^+^ and K^+^ ions upon membrane hyperpolarization; its gating kinetics are further modulated by intracellular cyclic nucleotides, most notably cyclic adenosine monophosphate (cAMP), which bind directly to the cyclic nucleotide-binding domain (CNBD) of the channel, shifting the voltage-dependence of activation toward more positive potentials and thereby increasing channel open probability (Porro et al., 2020). This cAMP-mediated facilitation of I_*f*_ represents the principal ionic mechanism through which sympathetic stimulation accelerates the rate of Phase four diastolic depolarization and, consequently, heart rate.

Pathogenic variants in HCN4, encompassing both loss-of-function mutations that reduce I_*f*_ amplitude or impair cAMP responsiveness, and dominant-negative mutations that disrupt normal channel trafficking or subunit assembly, compromise the automaticity of nodal pacemaker cells by attenuating the inward depolarizing current during Phase four. The resultant slowing or failure of spontaneous diastolic depolarization manifests clinically as a spectrum of bradyarrhythmias, most prominently sinus node dysfunction (SND) and AVB, reflecting the critical dependence of both SA and AV nodal automaticity on intact I_*f*_ function (Copier et al., 2025; Ntalla et al., 2020). The severity of the clinical phenotype is broadly proportional to the degree of I_*f*_ impairment: partial loss-of-function may present as isolated chronotropic incompetence or first-degree AVB, whereas complete or near-complete channel dysfunction may precipitate profound bradycardia, high-degree AVB, or syncope. Given that pharmacological options for augmenting I_*f*_ function in the context of HCN4 loss-of-function remain limited, patients with clinically significant SND or high-degree AVB attributable to HCN4 pathogenic variants are managed primarily through permanent pacemaker implantation, which provides reliable rate support independent of intrinsic nodal automaticity.

### GNB gene (G protein β subunit)

The GNB gene family (GNB1–5) demonstrates broad tissue expression, encompassing the heart, brain, and vasculature. Within the cardiac context, GNB expression is localized predominantly to cardiomyocytes and the CCS (De Nittis et al., 2021), where the encoded Gβ subunits assemble with Gα and Gγ subunits to form heterotrimeric G protein complexes. In the heart, Gβγ dimers exert essential electrophysiological regulatory functions by directly modulating key cardiac ion channels, independent of their conventional roles in cell proliferation and differentiation (Stott and Greenwood, 2024).

Specifically, the Gβγ dimer directly activates G protein-gated inwardly rectifying potassium (GIRK) channels, encoded by potassium inwardly rectifying channel subfamily J 3 (KCNJ3) and potassium inwardly rectifying channel subfamily J 5 (KCNJ5), thereby augmenting outward K^+^ conductance and promoting membrane hyperpolarization (Kano et al., 2019). Conversely, Gβγ exerts an inhibitory influence on both L-type voltage-dependent calcium channels (I_*Ca,L*_) and T-type calcium channels (I I_*Ca,T*_), attenuating inward Ca^2+^ current and consequently suppressing calcium-dependent membrane depolarization. The net electrophysiological consequence of Gβγ activation is therefore a shift toward hyperpolarization and reduced excitability, mediated through the simultaneous enhancement of repolarizing K^+^ efflux and suppression of depolarizing Ca^2+^ influx (Herman et al., 2026). Employing a paired GNB gene co-transfection strategy combined with whole-cell patch-clamp electrophysiology, Stallmeyer et al. systematically interrogated the relative contributions of these three channel targets and identified GIRK channel activation as the predominant mechanism underlying GNB mutation-associated AVB genesis (Stallmeyer et al., 2017), establishing GIRK-mediated hyperpolarization as the primary electrophysiological substrate of this conduction disorder. Consistent with this mechanistic framework, GNB is expressed in both the SA node and the AV node, the two nodal structures whose automaticity and conduction properties are most critically dependent on the precise balance of inward and outward ionic currents during Phase four diastolic depolarization. GOF mutations in GNB that constitutively or excessively activate cardiac GIRK channels induce pathological hyperpolarization of the resting membrane potential in SA and AV nodal cells, thereby raising the threshold for spontaneous Phase four depolarization, slowing the rate of impulse formation in the SA node, and impairing impulse propagation through the AV node (Fukuda et al., 2020). This dual impairment of pacemaker automaticity and atrioventricular conduction velocity provides a coherent electrophysiological basis for the bradyarrhythmias and varying degrees of AVB observed in patients harboring pathogenic GNB variants, and further highlights the GIRK channel axis as a potential therapeutic target in GNB-related conduction disease.

## Essential genetic disorders for cardiac malformation

**FIGURE 3:**
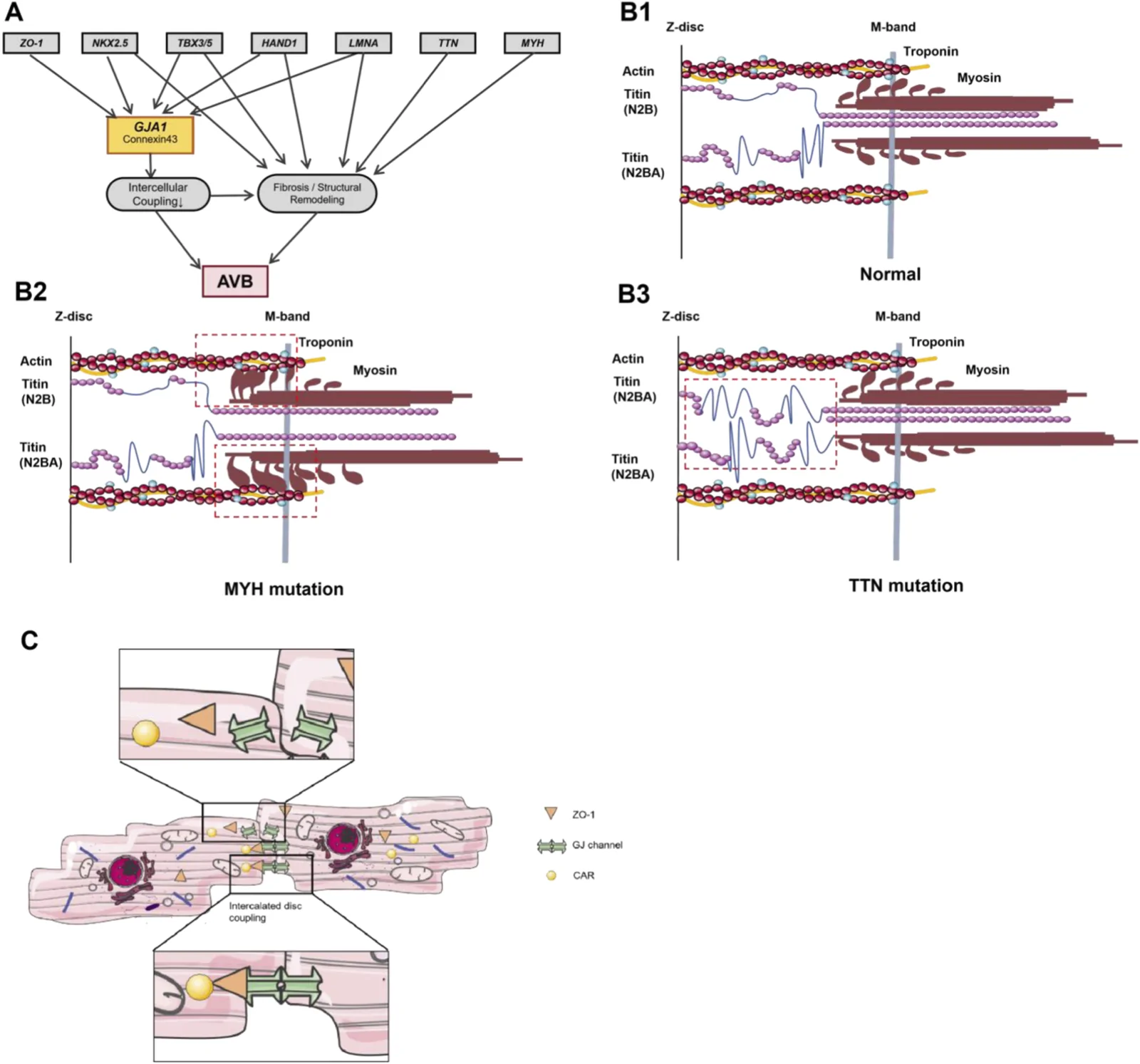
Schematic diagram of structure, cellular coupling and developmental mechanisms **(A)** The genes participate in cardiac development and morphology. Genes converge on two core pathogenic pathways: Impaired intercellular electrical coupling and myocardial fibrosis/structural remodeling, ultimately leading to AVB **(B)** The intracellular changes under *MYH* and *TTN* mutations. B1: Normal sarcomere structure with balanced titin isoforms (N2B/N2BA). B2: MYH mutations disrupt myosin autoinhibition, increasing the number of myosin heads available for actin binding, causing hypercontractility and myocardial hypertrophy. B3: TTN mutations shift the titin isoform ratio toward the compliant N2BA isoform, impairing sarcomere mechanical stability and promoting structural remodeling **(C)** ZO-1 binds to both GJ channels and CAR. In normal conditions, this complex stabilizes the membrane targeting, spatial organization, and retention of connexins and CAR, ensuring efficient electromechanical coupling between adjacent cardiomyocytes (Lower enlarged view). Disruption of ZO-1 diminishes CAR stability at the intercalated disc and promotes the disassembly of functional GJ complexes (Upper enlarged view).

Advances in genome-wide association studies (GWAS) and next-generation sequencing (NGS) technologies have substantially expanded the catalogue of gene loci implicated in cardiac conduction, extending the molecular landscape of AVB well beyond the canonical ion channelopathies discussed above. Notably, a distinct class of pathogenic variants has emerged in genes primarily governing cardiac structural integrity, specifically those encoding proteins essential for cytoskeletal architecture and intercellular connectivity of cardiomyocytes, that have been independently associated with the pathogenesis of AVB in the pediatric population. Unlike the ion channel-related mechanisms previously described, wherein conduction disturbances arise principally from direct electrophysiological dysfunction, these structurally oriented gene defects appear to disrupt atrioventricular conduction through the mechanical and organizational disruption of cardiomyocyte coupling, impaired gap junction function, and aberrant myocardial development, processes that are frequently co-expressed in the broader context of congenital heart disease (CHD). The following section examines the molecular basis by which mutations in genes governing cardiomyocyte structural connectivity and cardiac morphogenesis contribute to the pathogenesis of AVB in children (Figure 3).

### Gap junction genes

Gap junctions (GJs) are specialized intercellular communication structures present in select neuronal nuclei, vascular smooth muscle, and cardiac muscle, enabling the direct cell-to-cell propagation of action potentials and the rapid synchronization of electrical activity among functionally coupled cells (Sedovy et al., 2023; Kléber and Jin, 2021). Within the heart, three principal connexin isoforms constitute the structural basis of intercellular electrical coupling: Connexin 40 (CX40), Connexin 43 (CX43), and Connexin 45 (CX45), encoded by GJA5, GJA1, and GJC1, respectively (Guo and Yang, 2022). These isoforms exhibit a highly regionalized expression pattern that reflects their distinct electrophysiological roles across cardiac compartments. CX43 serves as the predominant gap junction protein mediating electrical coupling between adjacent ventricular myocytes, with additional expression in atrial myocytes and endothelial cells. CX40 is preferentially expressed in the atrial myocardium, where it contributes to the rapid conduction of atrial impulses. CX45, by contrast, is abundantly expressed during early embryonic myocardial development, where it is indispensable for normal cardiac morphogenesis, and in the mature heart is predominantly localized to the SA node, AV node, and Purkinje fiber network; loss of CX45 expression has been demonstrated to produce significant delays in atrioventricular conduction, underscoring its critical role in AV nodal electrophysiology (Zhang J. et al., 2020).

At the molecular level, gap junction channel biogenesis proceeds through the docking of two hemichannels (connexons), each comprising six connexin protomers contributed by adjacent cells, to form a complete intercellular channel. The assembled GJ channel permits the bidirectional exchange of ions and small molecules (typically <1 kDa) between neighboring cells, thereby mediating intercellular electrical coupling, metabolic coordination, and second messenger propagation. These functions are collectively essential for embryonic development, physiological homeostasis, and the coordinated regulation of tissue and organ function (Li et al., 2023; Garcia-Vega et al., 2021; Zhu, 2022; Zhang et al., 2021; Ai et al., 2021). Disruption of this intercellular communication architecture, whether through reduced connexin expression, impaired channel assembly, or compromised channel conductance—directly undermines the efficiency of electrophysiological signal transmission across the myocardium and the cardiac conduction system.

Pathogenic variants in GJC1 have been shown to exert specific and mechanistically informative effects on cardiac conduction system integrity. Structural and functional analyses of the CX45 (R184G) missense mutation revealed that this variant does not abolish connexin docking or prevent hemichannel assembly (Kléber and Jin, 2021). Rather, it selectively impairs the functional conductance of the assembled channel, reducing its ionic permeability and thereby attenuating intercellular electrical coupling without precluding channel formation (Li et al., 2023). In a separate line of investigation, GJC1 mutations were found to be associated with CHD, arrhythmogenic cardiomyopathy (ACM), and AVB, yet notably did not impair cardiomyocyte uptake of fluorescent dye tracers, a finding that dissociates gap junction protein downregulation from generalized cardiomyocyte dysfunction (Li et al., 2023). This observation suggests that the pathological consequence of GJC1 mutation is primarily a quantitative reduction in functional gap junction number at intercalated discs, rather than a global impairment of cardiomyocyte viability or metabolic activity (Chen et al., 2024). The resultant decrease in intercellular coupling efficiency diminishes the velocity and reliability of electrophysiological signal propagation across the AV conduction axis, providing a coherent mechanistic basis for the bradyarrhythmias and atrioventricular block observed in patients harboring GJC1 pathogenic variants. Collectively, these findings establish gap junction remodeling—mediated through both quantitative reduction and qualitative functional impairment of connexin expression, as a distinct and clinically significant molecular mechanism underlying AVB pathogenesis in the pediatric population.

### Tight junction genes

Tight junction-associated proteins have increasingly been recognized as critical determinants of cardiac conduction system integrity, with Zonula Occludens-1 (ZO-1) emerging as a significant regulatory factor in AVN function and disease (Dai et al., 2020). ZO-1 belongs to the membrane-associated guanylate kinase (MAGUK) protein superfamily, which encompasses three paralogous members, ZO-1, ZO-2, and ZO-3, characterized by conserved PDZ, SH3, and guanylate kinase-like (GUK) domains that mediate protein-protein interactions at cell junctions (Nielsen et al., 2023). At the cytoplasmic face of intercellular junctions, ZO-1 functions as a multivalent scaffolding protein bridging transmembrane junctional proteins to the cytoskeletal network, maintaining structural polarity, a role well-established in epithelial biology but now substantially expanded to encompass intercalated disc organization in the myocardium.

Within the cardiac context, ZO-1 directly interacts with filamentous actin, intercalated disc structural components, gap junction connexin proteins, and the Coxsackievirus and Adenovirus Receptor (CAR), a transmembrane protein implicated in intercalated disc organization and AV nodal conduction (Zhang J. et al., 2020; Dai et al. 2020; Liang and Sheikh, 2020). Through these interactions, ZO-1 coordinates the spatial organization, membrane targeting, and stabilization of gap junction complexes at the intercalated disc, directly regulating electromechanical coupling efficiency between adjacent cardiomyocytes. Cardiomyocyte-specific ablation of ZO-1 significantly reduces both the expression and intercalated disc localization of connexin proteins and CAR, demonstrating that ZO-1 actively governs connexin trafficking and membrane retention rather than serving as a passive structural scaffold. The consequent depletion of functional gap junction complexes broadly impairs intercellular electrical coupling, manifesting clinically as a spectrum of atrioventricular conduction disturbances ranging from first-degree AVB to complete heart block. These findings collectively establish ZO-1 dysfunction as a structurally driven and mechanistically distinct pathway to AVB pathogenesis, independent of primary ion channel dysfunction.

### Heart-specific transcription factor gene NKX2.5

NKX2.5, the first gene identified in association with atrial septal defect, encodes a homeodomain-containing transcription factor that serves as a master regulator of cardiac development (Cao et al., 2023). Its functional roles encompass cardiac morphogenesis, rightward cardiac looping, AV septation and specialization, and the long-term maintenance of the working cardiomyocyte conduction system. Structurally, NKX2.5 comprises three conserved functional domains: Tinman (TN) domain, the homeodomain (HD), and the NK2-specific domain (Cao et al., 2023). Although the precise function of the TN domain remains incompletely characterized, mutational studies have demonstrated that TN domain disruption results in failure of visceral mesoderm specification and primitive heart tube formation (Chung and Rajakumar, 2016), suggesting a role in the earliest stages of cardiogenic commitment. The HD domain constitutes the primary functional domain of NKX2.5, conferring sequence-specific DNA-binding capacity and transcriptional regulatory activity essential for downstream cardiac developmental gene programs.

Of particular relevance to AVB pathogenesis, aberrant overexpression of NKX2.5 has been shown to transcriptionally downregulate the expression of gap junction proteins CX43 and CX40 in cardiomyocytes, disrupting the molecular architecture of the AV node and His bundle and consequently impairing the coordinated propagation of electrical impulses through the AV conduction axis, manifesting as varying degrees of atrioventricular block (Ruiz-Pozo et al., 2025). Furthermore, NKX2.5 overexpression dysregulates cardiac α-actin expression, perturbing sarcomeric organization and cardiomyocyte contractile function, and promoting pathological myocardial hypertrophy (Schmidinger et al., 2008). Collectively, these findings demonstrate that NKX2.5 dosage dysregulation—through both loss- and gain-of-function mechanisms, disrupts the transcriptional networks governing conduction system development and cardiomyocyte structural integrity, establishing it as a pleiotropic contributor to AVB pathogenesis.

### T-box transcription factor gene TBX

The T-box (*TBX*) gene family encodes a phylogenetically conserved class of transcription factors that, analogous to the NKX family, play indispensable roles in embryonic development and organogenesis (Papaioannou, 2014). The family currently comprises 18 characterized members, of which TBX3 and TBX5 have been most extensively implicated in cardiac development and conduction system function. Aberrant TBX expression is associated with a broad spectrum of developmental and acquired diseases, reflecting the pleiotropic transcriptional programs these factors govern. TBX3 functions as a potent transcriptional repressor and is expressed at particularly high levels within the AV node relative to TBX5 (Anon (PDF)a). Mechanistically, TBX3 directly represses SCN5A, GJA1, and GJA5, thereby suppressing the fast-conduction gene program and maintaining the electrophysiological identity of the SA node, AV node, and His-Purkinje system (HPS) (Burnicka-Turek et al., 2025; Kumar et al., 2014; Basson et al., 1995). TBX3 haploinsufficiency causes Ulnar-Mammary syndrome, characterized by structural abnormalities of the upper limbs, mammary glands, and heart—with cardiac manifestations including myocardial fibrosis, cardiac enlargement, and atrioventricular block, collectively reflecting the loss of transcriptional maintenance of conduction system architecture. Conversely, TBX3 overexpression has been associated with oncogenesis and, within the cardiac context, with the induction of ectopic pacemaker foci and consequent arrhythmogenesis, underscoring the critical importance of precise TBX3 dosage regulation.

TBX5 plays an essential role in myocardial specification, cardiac septation, and conduction system formation. GWAS analyses have identified TBX5 variants as significantly associated with prolonged PR intervals, implicating it in AV conduction regulation (Su et al., 2017; Sinkovec et al., 2005; Burnicka-Turek et al., 2020). Mechanistically, TBX5 modulates AV conduction partly through transcriptional regulation of TBX3, establishing a hierarchical transcription factor axis governing conduction system integrity. Missense mutations in TBX5 underlie Holt-Oram syndrome, presenting with upper limb malformations and congenital cardiac defects, further demonstrating the convergent roles of TBX3 and TBX5 in AVB pathogenesis (Su et al., 2017; Burnicka-Turek et al., 2020).

### Cardiac neural spine derivatives express the transcription factor gene HAND

The Heart and Neural Crest Derivatives (HAND) gene family encodes basic helix-loop-helix (bHLH) transcription factors predominantly expressed in cardiomyocytes, comprising two paralogous members, HAND1 and HAND2, that fulfill complementary roles in cardiac and neural crest-derived tissue development (Vincentz et al., 2020; Firulli et al., 2025). Murine loss-of-function studies have established that HAND1 knockout produces severe cardiac phenotypes, including ventricular septal defect, double outlet right ventricle, and aortic override, while HAND1 overexpression is sufficient to cause sudden cardiac death, collectively demonstrating the critical importance of precise HAND1 dosage in maintaining cardiac structural integrity (Firulli et al., 2020). Beyond its developmental roles, HAND1 exerts direct regulatory influence over cardiac conduction through mechanistically distinct pathways. Vincentz et al. demonstrated that transgene-mediated overexpression of HAND1 in adult mice produced post-transcriptional downregulation of CX43, impairing gap junction-mediated intercellular electrical coupling (Firulli et al., 2025; Vincentz et al., 2019; Vincentz et al., 2020). Complementarily, Masuelli et al. identified a functional HAND1-β-catenin interaction, suggesting that HAND1 may indirectly destabilize CX43 through perturbation of the β-catenin signaling axis (Masuelli et al., 2020). These molecular disruptions collectively manifest as ventricular hypertrophy, progressive cardiac dilation, and significant prolongation of PR, QRS, and RR intervals, ultimately culminating in fatal arrhythmias. These findings establish aberrant HAND1 expression as a pleiotropic contributor to AVB pathogenesis, operating through both direct connexin dysregulation and indirect intercalated disc signaling disruption.

Besides, the HAND associated inhibitor of differentiation (ID) gene family encodes transcriptional repressors that lack intrinsic DNA-binding capacity, functioning through heterodimer formation with bHLH transcription factors to competitively inhibit their DNA binding, thereby broadly regulating cell differentiation and proliferation. Among the four characterized subtypes, ID2 has been most extensively implicated in cardiac conduction system development, operating cooperatively within a transcriptional network involving NKX2.5 and GATA4 to orchestrate conduction system specification and maturation. The mechanistic contributions of ID2 dysregulation to AVB pathogenesis are bidirectional. Ito et al. demonstrated that ID2 functions as a transcriptional repressor of voltage-gated calcium channel expression; under hyperaldosteronism, suppressed ID2 expression relieves calcium channel inhibition, promoting pathological electrophysiological remodeling manifesting as arrhythmias and myocardial hypertrophy (Ito et al., 2021). Conversely, ID2 overexpression reduces spontaneous action potential frequency in cardiomyocytes, independently precipitating arrhythmogenesis. Seyerle et al. further established through GWAS and animal model analyses that ID2 is significantly associated with PR interval prolongation, directly implicating it in AV conduction regulation (Ito et al., 2021; Moskowitz et al., 2011; Lim et al., 2008; Seyerle et al., 2018; Duffield et al., 2020). Mechanistically, ID2 modulates PR interval duration through transcriptional regulation of NKX2.5 and GATA4, and through modulation of light-induced Per1 expression within the circadian regulatory axis (Ward et al., 2010). Collectively, these findings establish ID2 dosage dysregulation as a pleiotropic determinant of AVB pathogenesis, converging on conduction system transcriptional networks and calcium channel homeostasis.

### Nuclear laminin gene

The *LMNA* gene encodes nuclear lamins A and C, which provide structural scaffolding to the nuclear envelope and play integral roles in cell division, transcriptional regulation, and DNA repair. Pathogenic *LMNA* mutations produce a broad spectrum of multisystem diseases, encompassing striated muscle disorders including dilated cardiomyopathy (DCM) and cardiac conduction system disease, lipodystrophy syndromes, peripheral neuropathy, and accelerated aging phenotypes (Macquart et al., 2019). Notably, *LMNA* mutations represent the second most common monogenic cause of DCM. Mechanistically, Cheedipudi et al. demonstrated that LMNA mutations disrupt the nuclear lamina network, rendering cardiomyocyte nuclei susceptible to rupture under contractile stress and triggering release of double-stranded DNA, which subsequently activates the cGAS-STING-TBK1-IRF signaling axis, inducing IFN-β and downstream pro-inflammatory mediators (Cheedipudi et al., 2022). This innate immune activation exacerbates myocardial fibrosis and cardiomyocyte apoptosis, culminating in ventricular dilation, contractile dysfunction, and progressive electrical conduction disturbances (An et al., 2024). Furthermore, *LMNA* mutations impair chromatin remodeling, disrupting the transcriptional regulation and membrane localization of cardiac ion channels, while concurrently reducing CX43 expression and intercalated disc distribution (Shah et al., 2021). The resultant gap junction remodeling impairs synchronized intercellular electrical propagation, prolonging action potential duration, slowing conduction velocity, and substantially elevating arrhythmogenic risk (Cheedipudi et al., 2022). Collectively, these findings establish *LMNA* mutations as mechanistically convergent drivers of AVB pathogenesis through structural nuclear disruption, innate immune activation, and connexin-mediated conduction system remodeling.

#### Encode the myonexin gene TTN

The *TTN* gene, one of the largest in the human genome, encodes titin, a giant sarcomeric protein indispensable for sarcomere assembly, mechanical sensing, and intracellular signaling (Stroik et al., 2024). Titin regulates sarcomere length homeostasis during both contraction and relaxation cycles, while simultaneously transducing multiple mechanosignaling pathways to modulate active sarcomeric contractility (Verdonschot et al., 2020). Pathogenic *TTN* variants therefore disrupt the fundamental biomechanical and signaling architecture of the cardiomyocyte. Mechanistically, Liu et al. identified the TTN variant p (Asn16429Lys) within the fibronectin type III domain—a modularly organized region implicated in integrin interactions, whereby disruption of integrin-mediated mechanotransduction directly impairs systolic myocardial function (Liu et al., 2020). GWAS analyses have further established significant associations between *TTN* variants and atrial and AV electrical activity, implicating titin dysfunction in conduction system pathophysiology (van Setten et al., 2018). At the isoform level, pathogenic *TTN* variants shift the titin isoform ratio toward the compliant N2BA isoform, paradoxically increasing myocardial passive stiffness, impairing length-dependent activation, and elevating diastolic filling pressures (Granzier and Labeit, 2025). This pathophysiological cascade constitutes a primary mechanistic driver of DCM and concurrently predisposes the myocardium to structural and electrophysiological remodeling, ultimately precipitating AVB (Loescher et al., 2021; Wang et al., 2025; Uniat et al., 2024). Collectively, these findings establish *TTN* variants as mechanistically convergent contributors to AVB pathogenesis through sarcomeric structural disruption, impaired mechanotransduction, and secondary myocardial remodeling.

### Myosin heavy chain gene MYH

The *MYH* gene family belongs to the myosin superfamily and encodes the heavy chain subunits that constitute the molecular motor components of myosin (Emami et al., 2025). This family is broadly classified into sarcomeric and non-sarcomeric myosin heavy chain genes; the former, predominantly expressed in skeletal muscle and myocardium, determines the contractile characteristics of muscle fibers and encompasses multiple subtypes including MYH1, MYH2, MYH4, MYH6, and MYH7, while the latter governs non-muscle cellular functions including cell motility and cytokinesis. Among these subtypes, *MYH7* is predominantly expressed in ventricular cardiomyocytes and represents the most clinically significant isoform in cardiac pathology (Gao et al., 2024). Pathogenic *MYH7* mutations shift the myosin molecule from its autoinhibited, sequestered conformation to a constitutively active state, increasing the proportion of myosin heads available for actin interaction (Sarkar et al., 2020). This dysregulated actomyosin cross-bridge cycling enhances myocardial contractility while simultaneously impairing relaxation kinetics, establishing the molecular basis for HCM. The resultant myocardial hypertrophy and architectural disarray create a structurally hostile substrate for normal cardiac electrical propagation (Ogawa et al., 2025).

Several pathological mechanisms have been proposed to account for AVB in the context of HCM. These include structural discontinuity within the His bundle conduction pathway, focal myocardial necrosis disrupting conduction tissue integrity, and hypertrophic thickening of small intramural coronary artery walls with consequent luminal narrowing, producing ischemic injury to the conduction system (Schiaffino, 2018; Yang et al., 2024; Zhang L. et al., 2020). Collectively, these findings establish *MYH7* mutations as mechanistically convergent inducers of AVB through sarcomeric hyperactivation, myocardial structural remodeling, and conduction system ischemic compromise.

## Metabolism dysfunction induces conductive disorder

**FIGURE 4:**
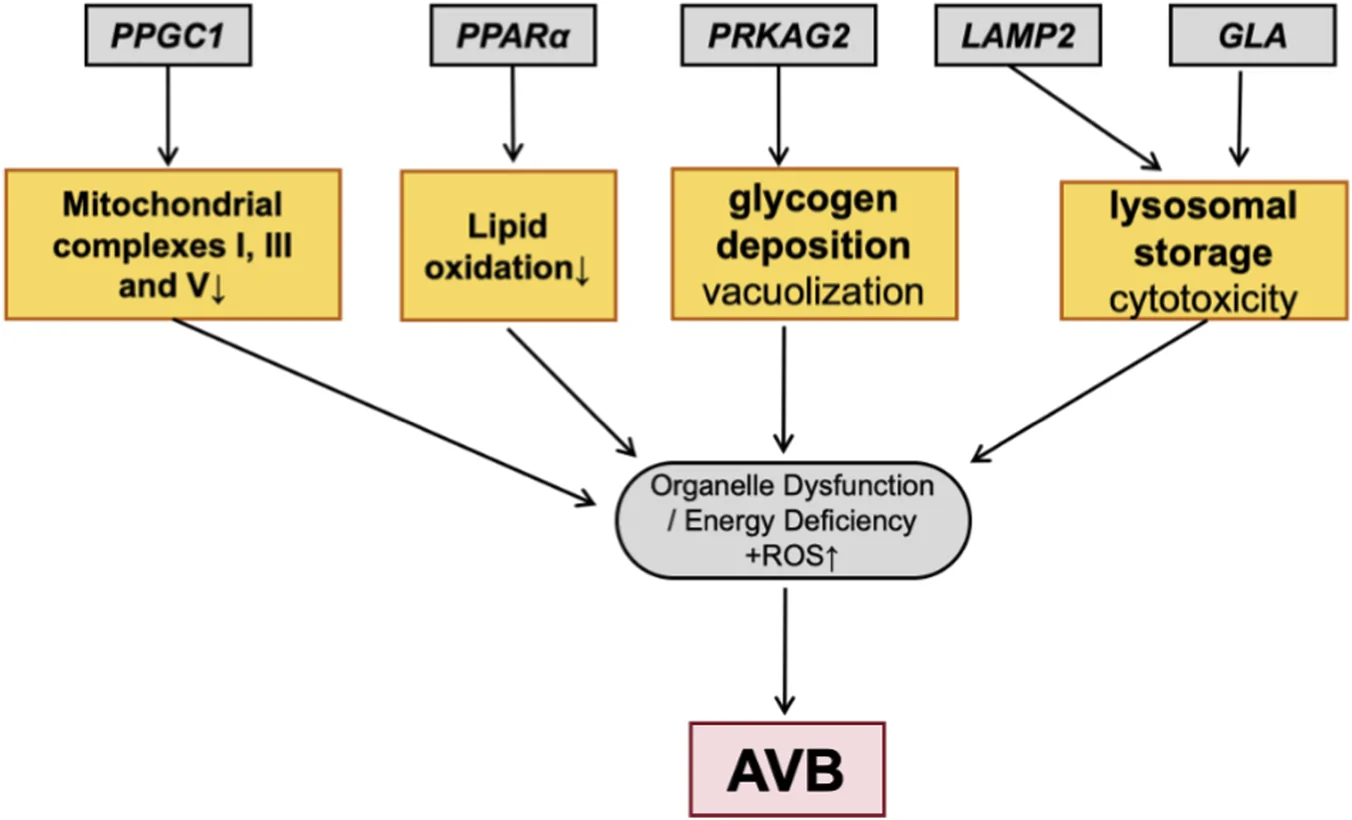
Schematic diagram of metabolic-organelle-electrophysiological imbalance mechanisms. Pathogenic variants in metabolism-related genes contribute to the development of AVB via distinct pathways. (1) PPGC1 impair mitochondrial respiratory chain function. (2) PPARα impair fatty acid oxidation. (2) PRKAG2 variant cause pathological glycogen deposition and vacuolization in cardiomyocytes. (3) LAMP2 and GLA variants induce lysosomal storage disorders and cytotoxicity. All these pathological changes disrupt myocardial energy metabolism homeostasis and redox equilibrium, ultimately disturbing electrophysiological signal transmission and aggravating cardiac conduction dysfunction.

Genes encoding metabolism-associated proteins exert both direct and indirect regulatory influence over the functional integrity of the cardiac conduction system, operating through interconnected mechanisms encompassing energy substrate utilization, oxidative stress homeostasis, and ionic equilibrium in cardiomyocytes. Under physiological conditions, cardiomyocytes predominantly rely on mitochondrial fatty acid β-oxidation as their primary energy substrate, generating the high-capacity ATP flux required to sustain continuous electromechanical activity. However, under pathophysiological stress conditions, including ischemia, hypoxia, and hemodynamic overload, cardiomyocytes undergo a metabolic substrate shift, transitioning toward anaerobic glycolysis as the predominant energy supply pathway. This metabolic reprogramming, while transiently adaptive, ultimately compromises ATP-dependent ion channel function, disrupts electrochemical gradients, and impairs action potential generation and propagation within the conduction system. Furthermore, dysregulated oxidative phosphorylation and accumulation of reactive oxygen species (ROS) inflict oxidative damage upon conduction tissue, exacerbating electrophysiological vulnerability. Pathogenic mutations in genes governing these metabolic axes therefore represent a mechanistically distinct yet clinically significant category of AVB determinants, converging on conduction system dysfunction through metabolic insufficiency rather than primary structural or ion channel pathology. The following section delineates key metabolism-related genes and their mechanistic contributions to cardiac conduction system dysfunction and AVB pathogenesis (Figure 4).

### AMP kinase encoding gene PRKAG2

*PRKAG2* encodes the γ2 regulatory subunit of AMP-activated protein kinase (AMPK), a master sensor of cellular energy homeostasis critically involved in metabolic regulation across multiple organ systems. Pathogenic PRKAG2 mutations constitutively activate AMPK signaling, precipitating a distinctive cardiac phenotype characterized by myocardial pseudohypertrophy and progressive conduction system dysfunction, frequently accompanied by systemic metabolic storage manifestations (Crea et al., 2026). Mechanistically, this pseudohypertrophic remodeling is fundamentally distinct from classical HCM, whereas HCM involves intrinsic cardiomyocyte disarray and myofibrillar architectural disruption, PRKAG2-associated hypertrophy arises exclusively from pathological glycogen accumulation within cardiomyocytes, expanding cellular volume without primary sarcomeric pathology, a distinction with significant therapeutic implications (Lopez-Sainz et al., 2020).

At the electrophysiological level, *PRKAG2* mutations exert multifaceted disruptive effects on cardiac ion channel function. Light et al. demonstrated that *PRKAG2* mutants induce delayed inactivation of voltage-gated sodium channels (L et al., 2003), prolonging depolarization and generating a pro-arrhythmic electrophysiological substrate. Concurrently, aberrant calcium channel function in *PRKAG2* mutant cardiomyocytes disrupts intracellular calcium cycling and impairs AV nodal conduction, directly precipitating AVB. The glycogen-laden conduction tissue further mechanically compromises the His-Purkinje system, amplifying conduction delay. These mechanistic insights have informed emerging therapeutic strategies: targeted reduction of pathological glycogen stores has demonstrated efficacy in attenuating disease progression in PRKAG2-associated cardiomyopathy. Furthermore, transgenic inhibition approaches in neonatal models have shown capacity to prevent aberrant accessory electrical pathway formation, mitigating downstream arrhythmogenic sequelae and conduction system deterioration.

### Peroxisome associated genes

Peroxisome proliferator-activated receptor alpha (PPARα) is a ligand-activated nuclear transcription factor belonging to the nuclear receptor superfamily, highly expressed in the myocardium where it serves as the master transcriptional regulator of cardiac fatty acid metabolism (Wang et al., 2021). Under physiological conditions, PPARα activation promotes heterodimerization with the retinoid x receptor (RXR), and the resulting heterodimeric complex binds to peroxisome proliferator response elements (PPREs) within the promoter regions of target genes, transcriptionally upregulating the fatty acid oxidation (FAO) machinery essential for sustaining myocardial ATP production (PPARα). This regulatory axis constitutes the predominant energy supply pathway in the healthy adult heart, where fatty acids account for approximately 60%–70% of total ATP generation. Pathological insufficiency of PPARα activation disrupts this metabolic equilibrium through a convergent series of molecular events. Downregulation of key FAO-related target genes, including carnitine palmitoyltransferase 1 A (CPT1A) and acyl-CoA oxidase 1 (ACOX1), impairs mitochondrial fatty acid import and peroxisomal β-oxidation capacity, respectively, resulting in the progressive intracellular accumulation of free fatty acids (FFA) and lipotoxic intermediates such as ceramides and diacylglycerols (Choi et al., 2024; Pparα, 2020). This lipotoxic milieu drives excessive mitochondrial reactive oxygen species (ROS) generation and activates pro-inflammatory signaling cascades, most notably the NF-κB pathway, which orchestrates the release of pro-inflammatory cytokines. The ensuing inflammatory microenvironment disrupts ionic homeostasis, compromises gap junction integrity and depletes the energy supply of the cardiac conduction system, collectively establishing a substrate for conduction disease and AVB. Paradoxically, under specific pathological conditions such as diabetic cardiomyopathy, PPARα overactivation produces a reciprocal lipotoxic phenotype. In this context, transcriptional upregulation of lipid uptake and synthesis pathways exceeds the oxidative capacity of the mitochondria, resulting in net myocardial lipid accumulation despite enhanced FAO gene expression, a pathological imbalance between lipid anabolism and catabolism that similarly impairs cardiomyocyte electromechanical function (Peng et al., 2022).

Beyond lipid metabolism, PPARα/RXR heterodimers exert significant regulatory influence over myocardial glucose utilization. The complex transcriptionally suppresses glucose transporter 4 (GLUT4) expression and downregulates glycolytic enzyme gene transcription, thereby reducing glucose uptake and glycolytic flux (Peng et al., 2022). Simultaneously, PPARα upregulates pyruvate dehydrogenase kinase 4 (PDK4) transcription, phosphorylating and inactivating the pyruvate dehydrogenase complex to inhibit glucose oxidation (Wright et al., 2009). This coordinated suppression of glucose metabolism renders cardiomyocytes metabolically inflexible, critically impairing their capacity to sustain ATP production during ischemic or hemodynamic stress when fatty acid availability is limited. The resultant bioenergetic insufficiency directly impairs ion channel function and action potential propagation within the conduction system, predisposing to AVB.

Peroxisome proliferator-activated receptor gamma coactivator-1 alpha (PGC-1α) is a pleiotropic transcriptional coactivator of multiple nuclear receptors, functioning as a central orchestrator of mitochondrial biogenesis, oxidative metabolism, and vascular homeostasis (Qian et al., 2024; Cao et al., 2025). Highly expressed in metabolically demanding tissues including the heart, skeletal muscle, liver, and kidneys, PGC-1α coordinates the transcriptional programs governing mitochondrial proliferation and respiratory chain assembly, and its dysregulation has been extensively implicated in type 2 diabetes mellitus and cardiovascular disease. At the molecular level, PGC-1α deficiency produces a cascade of mitochondrial dysfunction through transcriptional downregulation of mitochondrial respiratory chain complexes I, III, and V, critically impairing oxidative phosphorylation efficiency and precipitating myocardial energy depletion (Li et al., 2023). This bioenergetic deficit directly compromises the ATP-dependent maintenance of ion gradients essential for action potential generation, destabilizing depolarization, repolarization, and resting membrane potential with consequent electrophysiological dysregulation. Concurrently, mitochondrial dysfunction amplifies ROS generation while PGC-1α deficiency simultaneously reduces the transcriptional output of ROS-scavenging enzymes, including superoxide dismutase 2 (SOD2) and glutathione peroxidase 1 (GPX1), creating a state of uncompensated oxidative stress that inflicts oxidative damage upon conduction tissue and further destabilizes the electrophysiological regulatory network (Chadda et al., 2018; Ahmad et al., 2018). Beyond bioenergetic and redox dysregulation, PGC-1α deficiency impairs autonomic modulation of AV nodal conduction through transcriptional suppression of autonomic nervous system signaling genes, including adrenergic receptor alpha-1D (Adra1d) and adenylyl cyclase 4 (Adcy4). This attenuates the adaptive responsiveness of the AV node to neural regulatory inputs, disrupting the dynamic balance of conduction system modulation (Saadeh et al., 2021). The cumulative molecular insults converge to drive progressive myocardial structural remodeling and fibrosis, physically interrupting or delaying electrical signal propagation through the conduction system and ultimately precipitating atrioventricular block.

### Lysosome-related membrane protein gene LAMP2

*LAMP2* encodes lysosome-associated membrane protein 2, a transmembrane glycoprotein constitutively expressed on lysosomal membranes where it performs indispensable structural and functional roles in maintaining lysosomal integrity and mediating chaperone-assisted autophagy (Qiao et al., 2023). Pathogenic *LAMP2* mutations underlie Danon disease, an X-linked dominant lysosomal storage disorder whose cardinal molecular hallmark is a profound impairment of autophagic flux. Mechanistically, defective LAMP2 expression disrupts autolysosome formation precipitating the pathological intracellular accumulation of undigested glycogen granules and autophagic vacuoles within cardiomyocytes. This aberrant material accumulation drives progressive hypertrophic cardiomyopathy, skeletal myopathy, and variable degrees of intellectual disability (Ng et al., 2016). The resultant myocardial hypertrophy and vacuolar infiltration of conduction tissue mechanically disrupts electrical signal propagation, establishing a structural substrate for atrioventricular block and life-threatening arrhythmias. Critically, LAMP2 mutations confer a markedly elevated risk of sudden cardiac death, with conventional pacemaker implantation demonstrating limited efficacy in preventing fatal outcomes, underscoring the inadequacy of palliative electrophysiological interventions in the absence of molecular correction. Beyond cardiac transplantation, a Phase one clinical trial has demonstrated that intravenous infusion of RP-A501, a recombinant adeno-associated virus serotype 9 (AAV9) vector encoding the LAMP2B transgene, produced meaningful clinical improvement in Danon disease patients, representing a promising gene replacement therapeutic strategy (Yadin et al., 2023; Greenberg et al., 2025).

### Lysosomal enzyme gene GLA

*GLA* encodes α-galactosidase A (α-Gal A), a lysosomal hydrolase essential for glycosphingolipid (GSL) catabolism (Ortiz et al., 2018). Pathogenic GLA mutations reduce or completely ablate α-Gal A enzymatic activity, precipitating progressive lysosomal globotriaosylceramide (Gb3) accumulation, organelle swelling, and lysosomal membrane destabilization, collectively impairing cellular metabolic homeostasis and driving multi-organ dysfunction clinically manifesting as Fabry disease (FD) (Azevedo et al., 2021). Within the myocardium, pathological GSL deposition within cardiomyocytes and conduction tissue mechanically infiltrates and metabolically compromises the conduction system, manifesting as hypertrophic cardiomyopathy, atrioventricular block, and right bundle branch conduction disorders. The clinical trajectory of FD progresses from childhood-onset neuropathic pain and gastrointestinal manifestations toward fatal end-organ damage involving the kidneys, heart, and brain. Enzyme replacement therapy (ERT) remains the therapeutic cornerstone, with early intervention demonstrably preventing irreversible pathological progression (Ortiz et al., 2018; Politei et al., 2016). For eligible patients, oral migalastat represents a validated alternative therapeutic strategy.

## Immune disorders related AVB

### Maternal autoantibody-mediated mechanisms in fetal atrioventricular block

**FIGURE 5:**
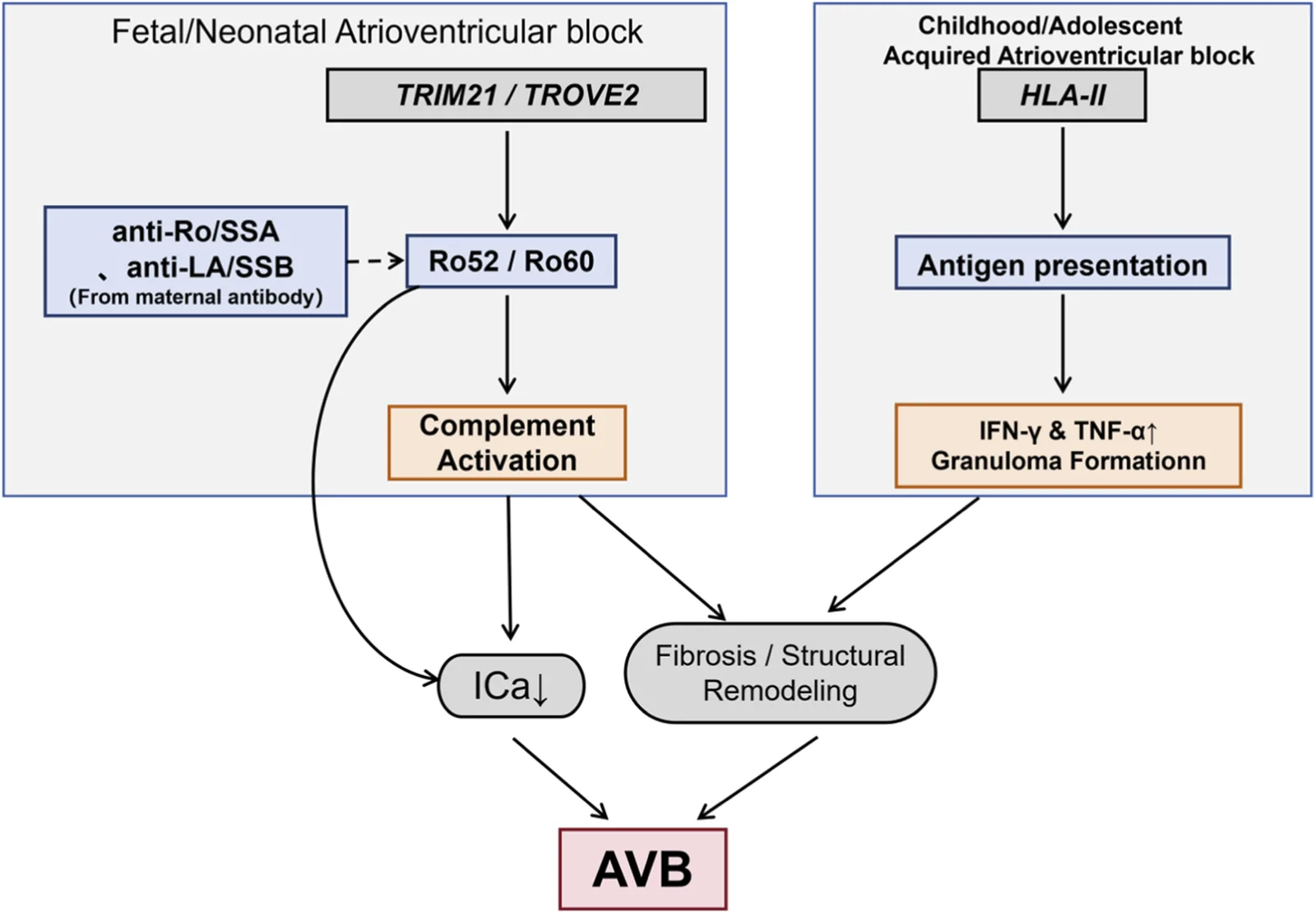
Schematic diagram of Immune-mediated mechanisms of Atrioventricular block. (1) Fetal/neonatal autoimmune AVB. Maternal anti-Ro/SSA and anti-La/SSB antibodies cross the placenta, targeting fetal conduction system antigens Ro52/TRIM21 and Ro60/TROVE2. They trigger complement activation and inflammation, while directly inhibiting L-type calcium currents. This dual insult causes fibrosis, structural remodeling, and AVB. (2) Childhood/adolescent acquired AVB. HLA-II variants promote aberrant antigen presentation, inducing pro-inflammatory cytokines, granuloma formation, and progressive fibrosis that disrupts the AV conduction axis, leading to acquired AVB.

Substantial clinical evidence from cumulative case reports has established a robust pathogenic association between fetal AVB and transplacental passage of maternal autoantibodies, predominantly anti-SSA/Ro and anti-SSB/La immunoglobulins, most frequently originating from mothers with systemic autoimmune conditions including systemic lupus erythematosus (SLE) and Sjögren’s syndrome (Ambrosi et al., 2021). Upon transplacental transfer into the fetal circulation, these autoantibodies selectively recognize and bind Ro52, Ro60, and La antigenic epitopes, which are preferentially overexpressed within the specialized cells of the fetal cardiac conduction system, most critically the AV node and the bundle of His. Antigen-antibody complex formation at these sites initiates complement system activation, precipitating a localized inflammatory cascade that inflicts progressive injury upon conduction tissue through cardiomyocyte apoptosis, inflammatory cell infiltration, and ultimately irreversible fibrotic replacement. This structural remodeling fundamentally disrupts ion channel function destabilizing action potential generation with propagation and establishing the electrophysiological substrate for conduction block (Figure 5).

### Molecular mechanisms of Anti-Ro52 antibody-induced conduction dysfunction

The principal autoantibody-encoding genes implicated in fetal autoimmune AVB are TRIM21 (encoding Ro52) and TROVE2 (encoding Ro60), whose aberrant antibody products constitute the primary molecular mediators of conduction system injury. Under physiological conditions, Ro52 — a RING-domain E3 ubiquitin ligase—functions as an interferon-inducible immunoregulatory protein that attenuates inflammatory signaling by targeting interferon regulatory factors 3 and 7 (IRF3/IRF7) for proteasomal degradation and suppressing NF-κB transcriptional activity (Panlu et al., 2025). Consequently, pathological anti-Ro52 autoantibody expression neutralizes this endogenous anti-inflammatory mechanism, disinhibiting inflammatory pathway activation and amplifying the pro-inflammatory microenvironment within fetal conduction tissue (Popescu et al., 2020). Anti-Ro52 antibodies are prevalent across a broad spectrum of systemic autoimmune diseases, including SLE, Sjögren’s syndrome, systemic sclerosis, scleroderma, rheumatoid arthritis, and inflammatory myopathies, and exert conduction system injury through two mechanistically distinct but synergistic pathways (Qu et al., 2019). First, anti-Ro52 antibodies directly bind the α1 subunit of L-type calcium channels (LTCC) in fetal cardiomyocytes, competitively inhibiting channel activity and reducing calcium ion influx. Given the fundamental dependence of AV nodal conduction on L-type calcium channel-mediated slow depolarization, this inhibition critically impairs both depolarization and repolarization kinetics, directly precipitating atrioventricular conduction block (Lazzerini et al., 2023). Second, anti-Ro52 antibodies potentiate cardiomyocyte apoptosis through a feed-forward inflammatory mechanism: during apoptotic cell death, intracellular Ro52 antigen undergoes translocation to the cardiomyocyte surface, becoming incorporated into apoptotic bodies. Surface-exposed Ro52 epitopes are subsequently recognized by circulating anti-Ro52 antibodies, further activating complement-mediated immune responses and perpetuating cycles of cardiomyocyte injury, inflammatory infiltration, and progressive fibrosis of the conduction system which induces AVB (Jones et al., 2021).

### HLA genetic variants and granulomatous conduction system disease

The human leukocyte antigen (HLA) gene complex encodes a family of cell surface glycoproteins that serve as the molecular foundation of adaptive immune recognition, functioning by presenting processed antigenic peptides to T lymphocytes to initiate antigen-specific immune responses. HLA class II molecules, encompassing HLA-DP, HLA-DQ, and HLA-DR, are expressed on professional antigen-presenting cells and present exogenously derived antigenic peptides to CD4^+^ helper T cells, thereby orchestrating humoral and cell-mediated immune activation. Eldhagen et al. identified a clinically significant association between specific HLA-DRB1 genotypes and cardiac granuloma formation in affected patients (Eldhagen et al., 2022). Mechanistically, HLA-DRB1 molecules present disease-specific antigenic peptides to CD4^+^ helper T cells, stimulating their clonal expansion and cytokine secretion, including TNF-α, IFN-γ, and IL-12, which collectively promote the recruitment and fusion of macrophages into multinucleated giant cells, driving granuloma formation and maintenance within the myocardium and conduction tissue. The resulting granulomatous infiltration of the AV node and infranodal conduction pathways produces progressive mechanical disruption of electrical signal transmission, manifesting clinically as second- and third-degree atrioventricular block.

## Other genes associated with AVB

### TNNI3K

*TNNI3K* encodes cardiac troponin I-interacting kinase, a cardiomyocyte-restricted serine/threonine kinase predominantly expressed in ventricular myocardium, where it phosphorylates cardiac troponin I to modulate myofilament calcium sensitivity and systolic contractile function. Pathological overexpression of TNNI3K disrupts normal myocardial contractility and, as demonstrated by Pham et al., suppresses connexin 45 (CX45) expression, a gap junction protein critically mediating intercellular electrical coupling between the AV node and surrounding cardiomyocytes (Pham et al., 2021). This CX45 downregulation impedes intercellular electrical signal transmission, mechanistically explaining the observed direct proportionality between TNNI3K expression levels and PR interval prolongation on electrocardiography. Furthermore, TNNI3K dysregulation perturbs voltage-gated calcium channel function, disrupting intracellular calcium homeostasis and further compromising conduction system integrity, collectively manifesting as AVB, bundle branch block, ventricular tachycardia, and ventricular fibrillation. Therapeutically, development of highly selective TNNI3K kinase inhibitors and CRISPR-based gene correction strategies represent promising interventional approaches to restore normal electrophysiological function.

### RNF207

*RNF207* encodes a RING domain-containing E3 ubiquitin ligase highly expressed in cardiomyocytes, exerting pleiotropic electrophysiological regulation through ubiquitin-proteasome-mediated post-translational modification of critical cardiac ion channel proteins (Yuan et al., 2023). Mechanistically, RNF207 stabilizes the cardiac voltage-gated sodium channel α-subunit SCN5A through targeted ubiquitination, maintaining physiological sodium current amplitude essential for rapid phase-0 depolarization and conduction velocity within the His-Purkinje system. Concurrently, RNF207 modulates cardiac repolarization through regulatory interactions with potassium channel subunits KCNQ1 and KCNH2, governing the slow and rapid components of the delayed rectifier potassium current (I_*Ks*_ and I_*Kr*_), respectively (Ledford et al., 2022). Additionally, RNF207-mediated regulation of CX43 ubiquitination governs gap junction turnover and intercellular electrical coupling efficiency, directly influencing conduction propagation velocity. Consequently, pathogenic RNF207 mutations simultaneously impair depolarization, repolarization, and intercellular coupling, producing a broad arrhythmogenic substrate encompassing LQTS and AVB.

## Future perspectives

### Complexity and intersectionality of genetic mechanisms underlying AVB

The genetic architecture of AVB is characterized by a paradigm of “polygenic convergence and multi-pathway intersectionality,” reflecting considerable mechanistic complexity and cross-regulatory interdependence. Ion channel genes, including *SCN5A, KCNH2*, and *TRPM4*, constitute the pathogenic core by directly modulating electrophysiological signaling. Among these, *SCN5A* occupies a central mechanistic position as the principal encoding gene for the cardiac voltage-gated sodium channel; loss-of-function mutations produce a phenotypic continuum ranging from isolated right bundle branch block to complete AVB, firmly establishing “channelopathy” as a fundamental etiological category in conduction system disorders. Gap junction protein genes (*GJA1, GJA5, GJC1*) and cardiac transcription factor genes (*NKX2.5, TBX3/5*) exert indirect but equally critical regulatory influence, while NKX2.5 could orchestrates both intercellular communication and embryonic conduction system morphogenesis. Notably, pathological overexpression of connexins CX43 and CX40 simultaneously downregulates gap junction conductance and disrupts sarcomere ultrastructure, exemplifying a “structure-function coupling” pathological mechanism wherein architectural and electrophysiological dysfunction are inextricably linked. Cross-pathway regulatory interactions further compound this complexity. The metabolic gene *PRKAG2* illustrates this principle compellingly: its gain-of-function mutations not only precipitate glycogen storage-associated myocardial hypertrophy but concurrently induce conduction block through delayed sodium channel inactivation kinetics, implicating a vicious cycle wherein metabolic dysregulation and ion channel dysfunction are mutually reinforcing through energy homeostasis imbalance. Similarly, immune-mediated mechanisms involving anti-Ro autoantibodies and HLA class II genes, while primarily propagating AVB through inflammatory tissue injury, converge upon shared terminal pathogenic pathways with non-immune mechanisms, including L-type calcium channel inhibition and gap junction disruption, underscoring the fundamental mechanistic unity underlying phenotypically diverse etiologies. All the mentioned genetic disorders were summarized in Table 1.

**TABLE 1:** The reported genes involved in AVBs.

Gene	Mutation	Effect of Mutation	Clinical Phenotype	Treatment	Country	Publication Year	DOI
SCN5A	5851G > T	V1951L	Vasovagal syncope Third-degree atrioventricular block	Modified vagal ganglion radio frequency ablation	China	2020	10.1186/s12887-020–02123–8
	4,850_4852delTCT	F1617del	Sudden cardiac death (SCD) Long-QT syndrome Cardiac conduction disease Brugada syndrome	​	Germany	2023	10.1016/j.hrthm. 2023.02.004
	3881C > G	A1294G	Atrioventricular II block	​	Russia	2019	10.1016/j.bbrc. 2019.06.080
	3823G > a	D1275N	First-degree atrioventricular block Complete right bundle branch block Sick sinus syndrome, Entricular tachycardia	Pacemaker implantation	Japan	2021	10.1007/s00795-021–00283–9
	1858C > T	R620C	Dilated cardiomyopathy (DCM) Single coronary artery anomaly	​	China	2023	10.3389/fcvm. 2023.1113886
	74 A > G	E25G	Dilated cardiomyopathy	Cardioverter defibrillator implantation	Spain	2021	10.3390/genes12121889
HERG	1838C > T	T613M	Left ventricular noncompaction Congenital long QT syndrome Type-2	Cardiac resynchronization therapy defibrillator implantation	Oman	2022	10.1053/j.jvca.2022.05.016
	1847A > G	Y616C	Left ventricular noncompaction combined with bradycardia Second-degree 2:1 atrioventricular block	​	China	2022	10.3389/fgene.2022.821226
	1764T > a	N588 K	Atrial fibrillation	​	Korea	2020	10.3346/jkms.2020.35.e238
	3140G > T	R1047L	Congestive heart failure Torsades de pointes	Dofetilide	United States of America	2004	10.1016/j.yjmcc.2004.09.001
​	1655T > C	L552S	Hereditary long QT syndrome type 2 2:1 atrioventricular block	Antiarrhythmic drugs Cardioverter defibrillator implantation	Finland	2000	10.1016/s0735-1097(00)00636-7
TRPM4	2455C > T	R819C	Inherited progressive cardiac conduction disease	Permanent pacemaker implantation	China	2021	10.1155/2021/9247541
	2351G > a	G844D	Complete atrioventricular block	Permanent pacemaker implantation	Italy	2022	10.3390/genes13020258
	583C > T	R195X	Short-coupled ventricular fibrillation Tachy-brady syndrome	Dual-chamber pacemaker and single-chamber ICD implantation	India	2025	10.1016/j.ipej.2025.03.003
HCN4	2648C > G	P883R	Atrial fibrillation	​	Germany	2019	10.1016/j.bbrc.2019.08.150
	3289G > T	G1097W	Complete atrioventricular block	Permanent pacemaker implantation	China	2014	10.1253/circj.cj-13-0996
	1590G > C	K530N	Familial tachycardia-bradycardia syndrome Persistent atrial fibrillation	Permanent pacemaker implantation	Germany	2013	10.1093/eurheartj/ehs391
	1631delC	P544fs	Sinus node dysfunction	Pacemaker implantation	Germany	2003	10.1172/JCI16387
	1444G > a	G482R	Biventricular noncompaction cardiomyopathy Sinus node dysfunction	Pacemaker implantation	Germany	2014	10.1016/j.jacc.2014.06.1155
GNB	155G > T	R52L	Autosomal dominant familial sinus node dysfunction Atrioventricular block	Permanent pacemaker implantation	Germany	2017	10.1161/CIRCRESAHA.116.310112
CX45	550 A > G	R184G	Autosomal dominant congenital heart disease Atrioventricular block	Surgical/catheter intervention Pacemaker implantation	China	2023	10.3390/biology12030346
	703 A > T	M235L	Atrial fibrillation	​	Canada	2021	10.1016/j.hrthm.2020.12.033
​	224G > a	R75H	Novel syndromic familial progressive atrioventricular block	Pacemaker implantation	France	2017	10.1016/j.jacc.2017.05.039
NKX2-5	437C > G	S146W	Familial idiopathic dilated cardiomyopathy	Pharmacological therapy Defibrillator implantation	China	2014	10.3892/ijmm. 2014.2029
	434T > C	F145S	Familial atrial fibrillation	Antiarrhythmic medications Catheter ablation Electrical cardioversion	China	2013	10.3892/ijmm.2013.1316
	424C > T	R142C	Atrial septal defect Atrioventricular block	Percutaneous interventional closure/surgical repair Antiarrhythmic medications	Canada	2017	10.1016/j.yjmcc.2017.03.003
	642C > T	T178M	Secundum atrial septal defect Hypoplastic left heart syndrome	Staged surgical reconstruction Permanent pacemaker implantation	Australia	2003	10.1016/s0735-1097(03)00420-0
TBX5	460T > G	S154 A	Idiopathic dilated cardiomyopathy	Pharmacological therapy Permanent pacemaker implantation	China	2015	10.1016/j.bbrc.2015.02.094
	394C > T	P132S	Atrial fibrillation Atypical holt–Oram syndrome	Permanent pacemaker implantation Percutaneous transcatheter closure or surgical repair	China	2016	10.3892/mmr. 2016.5043
	373G > a	G125R	Atypical holt–Oram syndrome Arrhythmias	Catheter radiofrequency ablation Permanent pacemaker implantation	Netherlands	2022	10.1161/CIRCULATIONAHA.121.054347
LMNA	349_350insG	K117fs	Atrioventricular block Dilated cardiomyopathy	Sunitinib, Sorafinib,Axitinib	United States of America	2019	10.1038/s41586-019-1406-x
	1621C > T	R541C	Dilated cardiomyopathy	​	China	2022	10.1016/j.ijcard.2022.06.038
	936G > C	Q312H	Atrioventricular block Atrial fibrillation	Permanent pacemaker implantation	China	2021	10.1161/CIRCGEN.119.00285
	665 A > C	H222P	Dilated cardiomyopathy	Simtuzumab	France	2026	10.1161/CIRCHEARTFAILURE.125.013806
	825_826insCAGG	A274fs	Atrioventricular block Atrial fibrillation	​	China	2021	10.1097/MD.0000000000025910
	936G > a	Q312Q	Dilated cardiomyopathy	Heart transplant	United States of America	2021	10.1002/ajmg.a.62530
TTN	98267C > T	T32756I	Paroxysmal atrial fibrillation	​	United States of America	2026	10.7554/eLife.104719
	87857G > a	W29286*	Left ventricular noncompaction, Arrhythmia	Pharmacological therapy	China	2023	10.1186/s12872-023-03382-w
	49287C > a	N16429 K	Atrioventricular block	Pacemaker implantation	China	2020	10.1016/j.ejmg.2019.103752
	33250G > a	E11084 K	Dilated cardiomyopathy Sudden cardiac death	Pharmacological therapy	United States of America	2024	10.1155/crig/9517735
MYH6	2161C > T	R721W	Coarctation of the aorta	Surgical repair	Iceland	2018	10.1093/eurheartj/ehy142
	539C > a	S180Y	Dilated cardiomyopathy Sudden cardiac death	Implantable cardioverter-defibrillator Pharmacological therapy	Italy	2019	10.1016/j.jelectrocard.2019.01.002
	2677G > a	V893M	Left ventricular noncompaction	Pharmacological treatment, implantable cardioverter-defibrillator	China	2026	10.2174/0113862073389079251128101041
PRKAG2	1591C > G	R531G	Wolff-Parkinson-White syndrome Immunoglobulin a nephropathy	Pharmacological therapy	United States of America	2019	10.1093/ehjcr/ytz038
​	911C > G	A304G	PRKAG2 cardiac syndrome Wolff-Parkinson-White syndrome	Radiofrequency ablation ECMO ICD implantation	United Kingdom	2020	10.1007/s00246-019-02245-6
	1681C > G	R531G	Wolff-Parkinson-White syndrome	Radiofrequency ablation Pharmacological therapy	United States of America	2001	10.1161/hc5001.102111
	298G > a	G100S	Dilated cardiomyopathy Sudden cardiac death	Septal ablation Pharmacological therapy	China	2016	10.3892/ijmm.2016.2565
LAMP2	928G > a	G310D	Danon disease	Heart transplantation	Greece	2019	10.1016/j.ejmg.2018.05.015
	925del	S309fs	Danon disease	Pharmacological therapy	United Kingdom	2,924	10.1080/13816810.2024.2404148
	573delA	K191fs	Danon disease	Pharmacological therapy	Japan	2009	10.1253/circj.cj-08-0241
GLA	937G > a	D313Y	Anderson–Fabry disease Left ventricular hypertrophy	Migalastat	Greece	2023	10.1093/ehjcr/ytad224
	427G > a	A143T	Fabry cardiomyopathy	Permanent pacemaker implantation, Pharmacological therapy	Finland	2020	10.1136/heartjnl-2019-315933
	70T > C	W24R	Fabry disease	Migalastat	Japan	2023	10.1016/j.ymgmr.2023.100982
	255 A > G	N419D	Fabry disease	Migalastat	Japan	2023	10.1016/j.ymgmr.2023.100982
TNNI3K	1577G > a	G526D	Cardiac conduction system disease Dilated cardiomyopath	Permanent pacemaker implantation Pharmacological therapy	United States of America	2014	10.1093/hmg/ddu297
	1534 A > C	I512L	Bundle branch re-entrant ventricular tachycardia	Implantable cardioverter-defibrillator	Spain	2025	10.1093/ehjcr/ytaf392

### Pleiotropic phenotypic characteristics of core pathogenic genes

A defining feature of AVB-associated genetic mutations is their capacity for “one cause, multiple effects”, wherein a single gene harbors mutation-site-dependent phenotypic diversity that may provide critical molecular clues for precision arrhythmia subclassification. *SCN5A* mutations exemplify this pleiotropy most strikingly: distinct mutation sites within the same gene confer divergent clinical phenotypes encompassing Brugada syndrome, long QT syndrome, and Lenègre disease, with phenotypic determination likely governed by the specific biophysical consequences of individual mutation sites on channel gating kinetics. Analogously, NKX2.5 functions as a transcriptional “master regulator” of cardiac development, wherein mutations may precipitate congenital AVB through transcriptional suppression of CX45 expression, or alternatively manifest as structural congenital heart disease through impaired cardiomyocyte proliferation and differentiation. Similarly, TBX3 haploinsufficiency underlies Ulnar-Mammary syndrome, featuring AVB and progressive myocardial fibrosis, whereas TBX3 overexpression paradoxically induces ectopic automaticity through aberrant pacemaker cell programming. The *ID2* gene further illustrates this complexity, as its dysregulated expression modulates PR interval duration through transcriptional regulation of NKX2.5 and GATA4, collectively suggesting that precise quantitative targeting of gene expression levels, rather than binary loss- or gain-of-function correction, will be essential for achieving precision therapeutic intervention in AVB.

### Translating mechanistic insights toward precision therapeutics

A conceptually important principle emerging from AVB genetics is the “bidirectional functional imbalance” of disease-associated genes, wherein both gain- and loss-of-function mutations within the same gene produce distinct but equally pathological phenotypes, necessitating mutation-specific rather than gene-specific therapeutic strategies. HCN4 exemplifies this duality: gain-of-function mutations impair sinus node automaticity through dysregulated hyperpolarization-activated funny current (I_*f*_), while loss-of-function mutations directly precipitate AVB through inadequate diastolic depolarization. For SCN5A loss-of-function-associated AVB, sodium channel activators represent a mechanistically rational pharmacological strategy to restore conduction velocity, whereas gain-of-function variants necessitate channel-blocking approaches. Likewise, *PRKAG2* gain-of-function mutations drive glycogen accumulation through pathological AMPK hyperactivation, while insufficient *PRKAG2* expression independently compromises myocardial energy metabolism motivating the development of “Goldilocks therapeutics” that restore physiological gene function within a precise activity window rather than simply augmenting or suppressing gene expression. Looking forward, gene editing technologies, particularly CRISPR-Cas9-mediated correction of pathogenic point mutations, and RNA interference strategies employing small interfering RNAs to suppress pathologically overexpressed targets such as *TNNI3K* represent transformative therapeutic modalities with potential for definitive molecular correction, transcending the palliative limitations of current electrophysiological interventions.

## Conclusion

The understanding of genetic mechanisms underlying pediatric atrioventricular block has undergone a paradigmatic transition, evolving from monogenic models toward an integrated framework of polygenic network dysregulation encompassing ion channelopathies, structural developmental defects, metabolic disorders, and immune-mediated conduction injury. This review has systematically delineated the complex molecular network governing AVB pathogenesis across these etiological categories, providing a comprehensive molecular foundation for mechanistic understanding and establishing a conceptual framework for precision clinical intervention. Nevertheless, significant limitations constrain current knowledge. First, the evidentiary basis for several gene-AVB associations remains restricted to small-cohort studies or isolated case reports, precluding robust genotype-phenotype correlation and necessitating large-scale prospective validation. Second, the unique biological characteristics of the pediatric population introduce additional complexity: neonatal AVB likely reflects dynamic interactions between maternal autoantibody exposure and fetal genetic susceptibility loci such as HLA-DRB1, yet existing investigations predominantly examine isolated etiological factors, inadequately accounting for “gene-environment” synergistic effects. Third, substantial genetic heterogeneity implicates modifier gene networks and epigenetic regulatory mechanisms as critical phenotypic determinants that remain incompletely characterized. Furthermore, the pathogenic contributions of non-coding genomic elements to AVB susceptibility remain substantially underexplored, representing a significant gap in current mechanistic understanding. Three priority research directions warrant focused future investigation. First, construction of pediatric-specific genetic reference databases incorporating age-stratified, sex-stratified, and developmental stage-stratified phenotypic data will be essential for delineating the complex interactions between developmental trajectory, sex-based biological differences, and genotype in determining conduction system disease expression. Second, systematic investigation of immune-genetic interaction mechanisms is imperative for developing composite biomarker panels capable of accurately stratifying fetal AVB risk in at-risk pregnancies. Third, the development of genotype-guided targeted therapeutics represents a clinically transformative objective that directly leverages mechanistic genetic insights.

In summary, while substantial progress has been achieved in elucidating the genetic underpinnings of pediatric AVB, the translational gap between gene discovery and clinical application remains considerable. Future advances predicated upon multidisciplinary collaboration, rigorous genotype-phenotype mapping, and integration of precision medicine technologies, encompassing next-generation sequencing, single-cell transcriptomics, and gene editing platforms, hold genuine promise for realizing early risk stratification, mechanistically informed diagnostic classification, and mutation-specific targeted therapy, ultimately improving long-term outcomes for children affected by this potentially life-threatening conduction disorder.

## References

[B2] Abu Rmilah A. A. Lin G. Begna K. H. Friedman P. A. Herrmann J. (2020). Risk of QTc prolongation among cancer patients treated with tyrosine kinase inhibitors. Int. J. Cancer 147, 3160–3167. 10.1002/ijc.33119 32449208 PMC8772338

[B7] Ahmad S. Valli H. Salvage S. C. Grace A. A. Jeevaratnam K. Huang C. L. (2018). Age‐dependent electrocardiographic changes in Pgc-1β deficient murine hearts. Clin. Exp. Pharmacol. Physiol. 45 (2), 174–186. 10.1111/1440-1681.12863 28949414 PMC5814877

[B3] Ai X. Yan J. Pogwizd S. M. (2021). Serine-threonine protein phosphatase regulation of Cx43 dephosphorylation in arrhythmogenic disorders *cell. Signal* . Cell. Signal. 86, 110070. 10.1016/j.cellsig.2021.110070 34217833 PMC8963383

[B4] Amarouch M.-Y. El Hilaly J. (2020). Inherited cardiac arrhythmia syndromes: focus on molecular mechanisms underlying TRPM4 channelopathies *cardiovasc. Ther* . Cardiovasc. Ther. 2020, 6615038. 10.1155/2020/6615038 33381229 PMC7759408

[B5] Ambrosi A. Thorlacius G. E. Sonesson S.-E. Wahren-Herlenius M. (2021). Interferons and innate immune activation in autoimmune congenital heart block. Scand. J. Immunol. 93, e12995. 10.1111/sji.12995 33188653

[B6] An C. Li Z. Chen Y. Huang S. Yang F. Hu Y. (2024). The cGAS-STING pathway in cardiovascular diseases: from basic research to clinical perspectives. Cell Biosci. 14, 58. 10.1186/s13578-024-01242-4 PMC11080250 38720328

[B40] Asatryan B. Medeiros-Domingo A. (2019). Molecular and genetic insights into progressive cardiac conduction disease. EP Eur. 21, 1145–1158. 10.1093/europace/euz109 31087102

[B41] Auricchio A. Demarchi A. Özkartal T. Campanale D. Caputo M. L. di Valentino M. (2023). Role of genetic testing in young patients with idiopathic atrioventricular conduction disease. Eur. Soc. Cardiol. 25, 643–650. 10.1093/europace/euac196 PMC9934995 36352534

[B42] Azevedo O. Cordeiro F. Gago M. F. Miltenberger-Miltenyi G. Ferreira C. Sousa N. (2021). Fabry disease and the heart: a comprehensive review. Int. J. Mol. Sci. 22 (9), 4434. 10.3390/ijms22094434 PMC8123068 33922740

[B18] Basson C. T. Solomon S. D. Weissman B. MacRae C. A. Poznanski A. K. Prieto F. (1995). Genetic heterogeneity of heart-hand syndromes. Circulation 91 (5), 1326–1329. 10.1161/01.cir.91.5.1326 7867169

[B43] Bello C. L. Mulay M. Huang X. Patyna S. Dinolfo M. Levine S. (2009). Electrocardiographic characterization of the QTc interval in patients with advanced solid tumors: Pharmacokinetic-pharmacodynamic evaluation of sunitinib. Clin. Cancer Res. 15, 7045–7052. 10.1158/1078-0432.CCR-09-1521 19903787

[B44] Bianchi B. Ozhathil L. C. Medeiros-Domingo A. Gollob M. H. Abriel H. (2018). Four TRPM4 cation channel mutations found in cardiac conduction diseases lead to altered protein stability. Front. Physiol. 9, 177. 10.3389/fphys.2018.00177 29568272 PMC5852105

[B46] Brink P. A. Ferreira A. Moolman J. C. Weymar H. W. van der Merwe P. L. Corfield V. A. (1995). Gene for progressive familial heart block type I maps to chromosome 19q13. Circulation 91, 1633–1640. 10.1161/01.cir.91.6.1633 7882468

[B47] Burnicka-Turek O. Broman M. T. Steimle J. D. Boukens B. J. Petrenko N. B. Ikegami K. (2020). Transcriptional patterning of the ventricular cardiac conduction system *circ. Res* . Circ. Res. 127, e94–e106. 10.1161/CIRCRESAHA.118.314460 32290757 PMC8328577

[B30] Burnicka-Turek O. Trampel K. A. Laforest B. Broman M. T. Yang X. H. Khan Z. (2025). Coordinated Tbx3/Tbx5 transcriptional control of the adult ventricular conduction system. bioRxiv. 2024.08.29.610377. 10.1101/2024.08.29.610377 PMC12289313 40705007

[B48] Cao C. Li L. Zhang Q. Li H. Wang Z. Wang A. (2023). Nkx2.5: a crucial regulator of cardiac development, regeneration and diseases. Front. Cardiovasc. Med. 10, 1270951. 10.3389/fcvm.2023.1270951 38124890 PMC10732152

[B32] Cao L. Li Y. Smirnov A. Voshtani R. Wang T. Shao C. (2025). PGC-1α: key regulator of mitochondrial biogenesis and cellular differentiation in metabolic and regenerative tissues. Cell Biosci. 16 (1), 9. 10.1186/s13578-025-01519-2 41456074 PMC12853593

[B17] Chadda K. R. Edling C. E. Valli H. Ahmad S. Huang C. L. Jeevaratnam K. (2018). Gene and protein expression profile of selected molecular targets mediating electrophysiological function in pgc-1α deficient murine atria. Int. J. Mol Sci. 19 (11), 3450. 10.3390/ijms19113450 30400228 PMC6274828

[B102] Cheedipudi S. M. Asghar S. Marian A. J. (2022). Genetic ablation of the DNA damage response pathway attenuates lamin-associated dilated cardiomyopathy in mice. JACC Basic Transl. Sci. 7 (12), 1232–1245. 10.1016/j.jacbts.2022.06.015 36644279 PMC9831927

[B49] Chen H. Li Y. X. Wong R. S. Esseltine J. L. Bai D. (2024). Genetically engineered human embryonic kidney cells as a novel vehicle for dual patch clamp study of human gap junction channels. Biochem. J. 481, 741–758. 10.1042/BCJ20240016 38752978 PMC11346430

[B31] Choi J. Smith D. M. Scafidi S. Riddle R. C. Wolfgang M. J. (2024). Carnitine palmitoyltransferase 1 facilitates fatty acid oxidation in a non-cell-autonomous manner. Cell Rep. 43 (12), 115006. 10.1016/j.celrep.2024.115006 PMC11726389 39671290

[B19] Chung I. M. Rajakumar G. (2016). Genetics of congenital heart defects: the NKX2-5 gene, a key player. Genes (Basel). 7 (2), 6. 10.3390/genes7020006 26805889 PMC4773750

[B50] Copier J. S. Verkerk A. O. Lodder E. M. (2025). HCN4 in the atrioventricular node heart rhythm. S1547–5271. 10.1016/j.hrthm.2025.02.030 39988103

[B51] Crea P. Moncada A. Catanzariti F. Agnelli G. Navarra M. Rubino C. (2026). Non-lysosomal glycogen storage cardiomyopathy with hypertrophic phenotype due to PRKAG2 c.905G>A. (p.Arg302Gln): case report and narrative review. Cardiogenetics 16 (2), 10.3390/cardiogenetics16010002

[B52] Dai W. Nadadur R. D. Brennan J. A. Smith H. L. Shen K. M. Gadek M. (2020). ZO-1 regulates intercalated disc composition and atrioventricular node conduction. Circ. Res. 127 e28–e43. 10.1161/CIRCRESAHA.119.316415 PMC7334106 32347164

[B53] Dong Y. Du R. Fan L.-L. Jin J.-Y. Huang H. Chen Y.-Q. (2021). Whole-exome sequencing identifies a novel TRPM4 mutation in a Chinese family with atrioventricular block. Biomed. Res. Int. 2021 2021, 9247541. 10.1155/2021/9247541 PMC8075657 33959666

[B54] Duffield G. E. Han S. Hou T. Y. de la Iglesia H. O. McDonald K. A. Mecklenburg K. L. (2020). 2020 inhibitor of DNA binding 2 (Id2) regulates photic entrainment responses in mice: differential responses of the Id2−/− mouse circadian system are dependent on circadian phase and on duration and intensity of light. J. Biol. Rhythms 35 555–575. 10.1177/0748730420957504 PMC9536081 32981454

[B55] Eldhagen P. Bobbio E. Darlington P. Grunewald J. Eklund A. Polte C. L. (2022). Phenotypic and HLA-DRB1 allele characterization of Swedish cardiac sarcoidosis patients. Int. J. Cardiol. 359, 108–112. 10.1016/j.ijcard.2022.04.006 35395284

[B27] Emami S. Mazloomi A. Ziadloo F. Hosseinzadeh S. Saeedi H. Khoshghiafeh A. (2025). MYH9: structure, functions, and therapeutic implications in cancer and genetic disorders. Genes Dis. 13 (4), 101977. 10.1016/j.gendis.2025.101977 PMC13054275 41953727

[B56] Firulli B. A. George R. M. Harkin J. Toolan K. P. Gao H. Liu Y. (2020). HAND1 loss-of-function within the embryonic myocardium reveals survivable congenital cardiac defects and adult heart failure. Cardiovasc. Res. 116, 605–618. 10.1093/cvr/cvz182 31286141 PMC7252443

[B29] Firulli B. A. Ferguson C. A. de Gier-de Vries C. Podicheti R. Rusch D. B. Christoffels V. M. (2025). Hand1 gene replacement with Hand2 reveals overlap in function with unique occurrence of omphalocele and heart defects. Development 152 (19), dev204963. 10.1242/dev.204963 PMC12587295 40960281

[B57] Fukuda T. Hiraide T. Yamoto K. Nakashima M. Kawai T. Yanagi K. (2020). Exome reports A *de novo* GNB2 variant associated with global developmental delay, intellectual disability, and dysmorphic features. Eur. J. Med. Genet. 63, 103804. 10.1016/j.ejmg.2019.103804 31698099

[B26] Gao Y. Peng L. Zhao C. (2024). MYH7 in cardiomyopathy and skeletal muscle myopathy. Mol. Cell Biochem. 479 (2), 393–417. 10.1007/s11010-023-04735-x 37079208

[B58] Garcia-Vega L. O’Shaughnessy E. M. Albuloushi A. Martin P. E. (2021). Connexins and the Epithelial Tissue Barrier: A Focus on Connexin 26 Biology. 10.3390/biology10010059 PMC7829877 33466954

[B12] Granzier H. L. Labeit S. (2025). Discovery of titin and its role in heart function and disease. Circ. Res. 136 (1), 135–157. 10.1161/CIRCRESAHA.124.323051 39745989

[B59] Greenberg B. Taylor M. Adler E. Colan S. Ricks D. Yarabe P. (2025). Phase 1 study of AAV9.LAMP2B gene therapy in danon disease. N. Engl. J. Med. 392, 972–983. 10.1056/NEJMoa2412392 39556016

[B60] Guo Y.-H. Yang Y.-Q. (2022). 2022 atrial fibrillation: focus on myocardial connexins and gap junctions biology. 11, 489. 10.3390/biology11040489 PMC9029470 35453689

[B61] Hennis K. Biel M. Fenske S. Wahl-Schott C. (2022). Paradigm shift: new concepts for HCN4 function in cardiac pacemaking. Pflugers Arch. 474, 649–663. 10.1007/s00424-022-02698-4 PMC9192375 35556164

[B62] Herman T. F. Cascella M. Muzio M. R. (2026). Mu Receptors Statpearls. Treasure Island FL: StatPearls Publishing. 31855381

[B63] Hu Y. Cang J. Hiraishi K. Fujita T. Inoue R. (2023). The role of TRPM4 in cardiac electrophysiology and arrhythmogenesis. Int. J. Mol. Sci. 24, 11798. 10.3390/ijms241411798 PMC10380800 37511555

[B64] Ito J. Minemura T. Wälchli S. Niimi T. Fujihara Y. Kuroda S. (2021). Id2 represses aldosterone-stimulated cardiac T-Type calcium channels expression. Int. J. Mol. Sci. 22, 3561. 10.3390/ijms22073561 PMC8037527 33808082

[B65] Jones E. L. Laidlaw S. M. Dustin L. B. (2021). TRIM21/Ro52 - roles in innate immunity and autoimmune disease. Front. Immunol. 12, 738473. 10.3389/fimmu.2021.738473 PMC8450407 34552597

[B36] Kano H. Toyama Y. Imai S. Iwahashi Y. Mase Y. Yokogawa M. (2019). Structural mechanism underlying G protein family-specific regulation of G protein-gated inwardly rectifying potassium channel. Nat. Commun. 10 (1), 2008. 10.1038/s41467-019-10038-x 31043612 PMC6494913

[B66] Kléber A. G. Jin Q. (2021). Coupling between cardiac cells—An important determinant of electrical impulse propagation and arrhythmogenesis. Biophys. Rev. 2, 031301. 10.1063/5.0050192 PMC8281002 34296210

[B11] Kumar P. P Emechebe U. Smith R. Franklin S. Moore B. Yandell M. (2014). Coordinated control of senescence by lncRNA and a novel T-box3 co-repressor complex. Elife 3, e02805. 10.7554/eLife.02805 24876127 PMC4071561

[B1] Lazzerini P. E. Murthy Ginjupalli V. K. Srivastava U. Bertolozzi I. Bacarelli M. R. Verrengia D. (2023). Anti-Ro/SSA antibodies blocking calcium channels as a potentially reversible cause of atrioventricular block in adults. JACC Clin. Electrophysiol. 9 (8 Pt 3):1631–1648. 10.1016/j.jacep.2023.03.007 37227349

[B67] Light P. E. Wallace C. H. R. Dyck J. R. B. (2003). Constitutively active adenosine monophosphate-activated protein kinase regulates voltage-gated sodium channels in ventricular myocytes. Circulation 107, 1962–1965. 10.1161/01.CIR.0000069269.60167.02 12682004

[B68] Ledford H. A. Ren L. Thai P. N. Park S. Timofeyev V. Sirish P. (2022). Disruption of protein quality control of the human *ether-à-go-go Related* gene K+ channel results in profound long QT syndrome *heart rhythm* . Heart rhythm. 19, 281–292. 10.1016/j.hrthm.2021.10.005 PMC8810706 34634443

[B69] Li Y.-J. Wang J. Ye W. G. Liu X.-Y. Li L. Qiu X.-B. (2023). Discovery of GJC1 (Cx45) as a new gene underlying congenital heart disease and arrhythmias biology. 10.3390/biology12030346 PMC10045734 36979038

[B70] Li B. Xu H. Wu L. (2025). Genetic insights into cardiac conduction disorders from genome-wide association studies *hum. Genomics* . 10.1186/s40246-025-00732-x PMC11871809 40022259

[B39] Liang Y. Sheikh F. (2020). Zippering up a role for ZO-1 in atrioventricular node conduction and disease. Circ. Res. 127 (2), 298-300. 10.1161/CIRCRESAHA.120.317291 32614716 PMC8204722

[B22] Lim J. Y. Kim W. H. Kim J. Park S. I. (2008). Induction of Id2 expression by cardiac transcription factors GATA4 and Nkx2.5. J. Cell. Biochem. 103 (1), 182–194. 10.1002/jcb.21396 17559079

[B16] Liu H. El Zein L. Kruse M. Guinamard R. Beckmann A. Bozio A. (2010). Gain-of-function mutations in TRPM4 cause autosomal dominant isolated cardiac conduction disease. Circ. Cardiovasc. Genet. 3 (4), 374–385. 10.1161/CIRCGENETICS.109.930867 20562447

[B71] Liu G. Yang Z. Chen W. Xu J. Mao L. Yu Q. (2020). Novel missense variant in TTN cosegregating with familial atrioventricular block. Eur. J. Med. Genet. 63, 103752. 10.1016/j.ejmg.2019.103752 31470098

[B72] Loescher C. M. Hobbach A. J. Linke W. A. (2021). Titin (TTN): from molecule to modifications, mechanics, and medical significance *cardiovasc* . Res 118, 2903–2918. 10.1093/cvr/cvab328 PMC9648829 34662387

[B73] Lopez-Sainz A. Dominguez F. Lopes L. R. Ochoa J. P. Barriales-Villa R. Climent V. (2020). Clinical features and natural history of PRKAG2 variant cardiac glycogenosis. J. Am. Coll. Cardiol. 76, 186–197. 10.1016/j.jacc.2020.05.029 32646569

[B74] Macquart C. Jüttner R. Morales Rodriguez B. Le Dour C. Lefebvre F. Chatzifrangkeskou M. (2019). Microtubule cytoskeleton regulates connexin 43 localization and cardiac conduction in cardiomyopathy caused by mutation in A-type lamins gene *hum. Mol* . Genet 28, 4043–4052. 10.1093/hmg/ddy227 29893868

[B75] Masuelli L. Bei R. Sacchetti P. Scappaticci I. Francalanci P. Albonici L. (2020). P β-Catenin Accumulates in Intercalated Disks of Hypertrophic Cardiomyopathic Hearts. 10.1016/j.cardiores.2003.08.005 14613867

[B76] Milner A. Mitrani L. R. Ferrara L. Lala A. Shaw L. J. Love B. (2024). Fetal arrhythmia leading to a diagnosis of congenital long QT syndrome type II *JACC case rep* . JACC. Case Rep. 29, 102218. 10.1016/j.jaccas.2023.102218 38379654 PMC10874987

[B77] Moore J. P. Gallotti R. G. Shannon K. M. Bos J. M. Sadeghi E. Strasburger J. F. (2020). Genotype predicts outcomes in fetuses and neonates with severe congenital long QT syndrome. JACC Clin. Electrophysiol. 6, 1561–1570. 10.1016/j.jacep.2020.06.001 33213816 PMC7679474

[B38] Moskowitz I. P. Wang J. Peterson M. A. Pu W. T. Mackinnon A. C. Oxburgh L. (2011). Transcription factor genes Smad4 and Gata4 cooperatively regulate cardiac valve development. Proc. Natl. Acad. Sci. U S A. 108 (10), 4006–4011. 10.1073/pnas.1019025108 21330551 PMC3053967

[B8] Ng K. M. Mok P. Y. Butler A. W. Ho J. C. Choi S. W. Lee Y, K. (2016). Amelioration of X-linked related autophagy failure in danon disease with DNA methylation inhibitor. Circulation 134 (18), 1373–1389. 10.1161/CIRCULATIONAHA.115.019847 27678261

[B78] Nielsen M. S. van Opbergen C. J. M. van Veen T. A. B. Delmar M. (2023). The intercalated disc: a unique organelle for electromechanical synchrony in cardiomyocytes. Physiol. Rev. 103, 2271–2319. 10.1152/physrev.00021.2022 36731030 PMC10191137

[B79] De Nittis P. Efthymiou S. Sarre A. Guex N. Chrast J. Putoux A. (2021). Inhibition of G-protein signalling in cardiac dysfunction of intellectual developmental disorder with cardiac arrhythmia (IDDCA) syndrome *J. Med* . Genet 58, 815–831. 10.1136/jmedgenet-2020-107015 PMC8639930 33172956

[B80] Nordenswan H.-K. Pöyhönen P. Lehtonen J. Ekström K. Uusitalo V. Niemelä M. (2022). Incidence of sudden cardiac death and life-threatening arrhythmias in clinically manifest cardiac sarcoidosis with and without current indications for an implantable cardioverter defibrillator. Circulation 146, 964–975. 10.1161/CIRCULATIONAHA.121.058120 PMC9508990 36000392

[B81] Ntalla I. Weng L.-C. Cartwright J. H. Hall A. W. Sveinbjornsson G. Tucker N. R. (2020). Multi-ancestry GWAS of the electrocardiographic PR interval identifies 202 loci underlying cardiac conduction. Nat. Commun. 11, 2542. 10.1038/s41467-020-15706-x 32439900 PMC7242331

[B82] Ogawa Y. Mizuno Y. Kato S. Shiraga K. Masuda H. Kondo A. (2025). A novel MYH7 lever region mutation causes infantile-onset restrictive cardiomyopathy with atrioventricular block and right ventricle hypoplasia *can* . J. Cardiol. 41, 2105–2108. 10.1016/j.cjca.2025.05.015 40441299

[B83] Ortiz A. Germain D. P. Desnick R. J. Politei J. Mauer M. Burlina A. (2018). Fabry disease revisited: management and treatment recommendations for adult patients. Mol. Genet. Metab. 123, 416–427. 10.1016/j.ymgme.2018.02.014 29530533

[B84] Palladino A. Papa A. A. Petillo R. Scutifero M. Morra S. Passamano L. (2022). The role of TRPM4 gene mutations in causing familial progressive cardiac conduction disease: a further contribution. Genes 258, 258. 10.3390/genes13020258 PMC8871839 35205305

[B15] Panlu K. Wang Y. Zhu X. Lin Z. Fang T. Yao X. (2025). TRIM21 regulation of IRF-mediated type I interferon signaling in systemic autoimmune diseases. Front. Immunol. 16, 1695818. 10.3389/fimmu.2025.1695818 41383611 PMC12689539

[B85] Peng M. Fu Y. Wu C. Zhang Y. Ren H. Zhou S. (2022). Signaling pathways related to oxidative stress in diabetic cardiomyopathy. Front. Endocrinol. 13, 907757. 10.3389/fendo.2022.907757 PMC9240190 35784531

[B37] Papaioannou V. E. (2014). The T-box gene family: emerging roles in development, stem cells and cancer. Development 141 (20), 3819–3833. 10.1242/dev.104471 25294936 PMC4197708

[B86] Pham C. Muñoz-Martín N. Lodder E. M. (2021). The diverse roles of TNNI3K in cardiac disease and potential for treatment. Int. J. Mol. Sci. 22, 6422. 10.3390/ijms22126422 34203974 PMC8232738

[B87] Politei J. M. Bouhassira D. Germain D. P. Goizet C. Guerrero-Sola A. Hilz M. J. (2016). Pain in fabry disease: practical recommendations for diagnosis and treatment *CNS neurosci. Ther* . CNS Neurosci. Ther. 22, 568–576. 10.1111/cns.12542 27297686 PMC5071655

[B88] Popescu M. R. Dudu A. Jurcut C. Ciobanu A. M. Zagrean A.-M. Panaitescu A. M. (2020). A broader perspective on Anti-Ro antibodies and their fetal consequences—A case report and literature review diagnostics 10, 478. 10.3390/diagnostics10070478 PMC7399931 32674462

[B33] Proost V. M. van den Berg M. P. Remme C. A. Wilde A. A. M. (2023). SCN5A-1795insD founder variant: a unique Dutch experience spanning 7 decades. Neth. Heart J. 31 (7–8), 263-271. 10.1007/s12471-023-01799-8 37474841 PMC10400486

[B89] Porro A. Thiel G. Moroni A. Saponaro A. (2020). Cyclic AMP regulation and its command in the pacemaker channel HCN4. Front. Physiol. 11, 771. 10.3389/fphys.2020.00771 32733276 PMC7358946

[B21] Qi M. Ma S. Liu J. Liu X. Wei J. Lu W. J. (2024). In Vivo base editing of scn5a rescues type 3 long QT syndrome in mice. Circulation 149 (4), 317-329. 10.1161/CIRCULATIONAHA.123.065624 37965733

[B90] Pparα A. (2020). Linking Cardiac Metabolism to Therapeutic Opportunities in Cardiovascular Diseases. 10.3390/cells15100940 PMC13204005 42193950

[B91] Qian L. Zhu Y. Deng C. Liang Z. Chen J. Chen Y. (2024). Peroxisome proliferator-activated receptor gamma coactivator-1 (PGC-1) family in physiological and pathophysiological process and diseases. Signal Transduct. Target. Ther. 9, 50. 10.1038/s41392-024-01756-w 38424050 PMC10904817

[B23] Qiao L. Hu J. Qiu X. Wang C. Peng J. Zhang C. (2023). LAMP2A, LAMP2B and LAMP2C: similar structures, divergent roles. Autophagy 19 (11), 2837–2852. 10.1080/15548627.2023.2235196 37469132 PMC10549195

[B92] Qu Y. S. Lazzerini P. E. Capecchi P. L. Laghi-Pasini F. El Sherif N. Boutjdir M. (2019). Autoimmune calcium channelopathies and cardiac electrical abnormalities. Front. Cardiovasc. Med. 6, 54. 10.3389/fcvm.2019.00054 31119135 PMC6507622

[B93] Remme C. A. (2013). Cardiac sodium channelopathy associated with SCN5A mutations: electrophysiological, molecular and genetic aspects. J. Physiol. 591, 4099–4116. 10.1113/jphysiol.2013.256461 23818691 PMC3779105

[B34] Remme C. A. (2023). SCN5A channelopathy: arrhythmia, cardiomyopathy, epilepsy and beyond. Philos. Trans. R Soc. Lond. B Biol. Sci. 378 (1879), 20220164. 10.1098/rstb.2022.0164 37122208 PMC10150216

[B94] Ripoll-Vera T. Zorio E. Gámez J. M. Molina P. Govea N. Crémer D. (2015). Phenotypic patterns of cardiomyopathy caused by mutations in the desmin gene. A clinical and genetic study in two inherited heart disease units. Rev. Esp. Cardiol. 68, 1027–1029. 10.1016/j.rec.2015.07.007 26431784

[B95] Ruiz-Pozo V. A. Cadena-Ullauri S. Paz-Cruz E. Tamayo-Trujillo R. Guevara-Ramirez P. Onofre-Ruiz P. (2025). Identification of a novel NKX2-5 variant in a young Ecuadorian patient with atrioventricular block and bradycardia: a case report. Front. Cardiovasc. Med. 12, 1552423. 10.3389/fcvm.2025.1552423 40255339 PMC12006191

[B96] Saadeh K. Chadda K. R. Ahmad S. Valli H. Nanthakumar N. Fazmin I. T. (2021). Molecular basis of ventricular arrhythmogenicity in a Pgc-1α deficient murine model. Mol. Genet. Metab. Rep. 27, 100753. 10.1016/j.ymgmr.2021.100753 PMC8059080 33898262

[B97] Sarkar S. S. Trivedi D. V. Morck M. M. Adhikari A. S. Pasha S. N. Ruppel K. M. (2020). The hypertrophic cardiomyopathy mutations R403Q and R663H increase the number of myosin heads available to interact with actin. Sci. Adv. 6 (eaax0069), eaax0069. 10.1126/sciadv.aax0069 32284968 PMC7124958

[B10] Schmidinger M. Zielinski C. C. Vogl U. M. Bojic A. Bojic M. Schukro C. (2008). Cardiac toxicity of sunitinib and sorafenib in patients with metastatic renal cell carcinoma. J. Clin. Oncol. 26 (32), 5204–5212. 10.1200/JCO.2007.15.6331 18838713

[B98] Schiaffino S. (2018). Muscle fiber type diversity revealed by anti-myosin heavy chain antibodies. FEBS J. 285, 3688–3694. 10.1111/febs.14502 29761627

[B99] Sedovy M. W. Leng X. Leaf M. R. Iqbal F. Payne L. B. Chappell J. C. (2023). Connexin 43 across the vasculature: gap junctions and beyond *J. Vasc. Res* . J. Vasc. Res. 60, 101–113. 10.1159/000527469 36513042 PMC11073551

[B20] Seyerle A. A. Lin H. J. Gogarten S. M. Stilp A. Méndez Giráldez R. Soliman E. (2018). Genome-wide association study of PR interval in Hispanics/Latinos identifies novel locus at ID2. Heart 104 (11), 904–911. 10.1136/heartjnl-2017-312045 29127183 PMC6946379

[B28] Shah P. P. Lv W. Rhoades J. H. Poleshko A. Abbey D. Caporizzo M. A. (2021). Pathogenic LMNA variants disrupt cardiac lamina-chromatin interactions and de-repress alternative fate genes. Cell Stem. Cell 28 (5), 938–954.e9. 10.1016/j.stem.2020.12.016 33529599 PMC8106635

[B13] Sinkovec M. Petrovic D. Volk M. Peterlin B. (2005). Familial progressive sinoatrial and atrioventricular conduction disease of adult onset with sudden death, dilated cardiomyopathy, and brachydactyly. a new type of heart-hand syndrome? Clin. Genet. 68 (2), 155–160. 10.1111/j.1399-0004.2005.00476.x 15996213

[B14] Stroik D. Gregorich Z. R. Raza F. Ge Y. Guo W. (2024). Titin: roles in cardiac function and diseases. Front. Physiol. 15, 1385821. 10.3389/fphys.2024.1385821 38660537 PMC11040099

[B101] Shin J. A. Choi Y. U. Kim K. M. Yoon J. H. Lee J. Y. (2021). Congenital long QT syndrome type 2 with symptomatic 2:1 atrioventricular block and ventricular arrhythmia in a preterm baby who presented with fetal ventricular tachycardia and hydrops. Korean Circ. J. 51, 792–796. 10.4070/kcj.2021.0202 PMC8424453 34494407

[B103] Stallmeyer B. Kuß J. Kotthoff S. Zumhagen S. Vowinkel K. Rinné S. (2017). A mutation in the G-Protein gene GNB2 causes familial sinus node and atrioventricular conduction dysfunction. Circ. Res. 120, e33–e44. 10.1161/CIRCRESAHA.116.310112 28219978

[B104] Stott J. B. Greenwood I. A. (2024). G protein βγ regulation of KCNQ-Encoded voltage-dependent K channels. Front. Physiol. 15, 1382904. 10.3389/fphys.2024.1382904 38655029 PMC11035767

[B105] Su W. Zhu P. Wang R. Wu Q. Wang M. Zhang X. (2017). Congenital heart diseases and their association with the variant distribution features on susceptibility genes. Clin. Genet. 91, 349–354. 10.1111/cge.12835 27426723

[B106] Surget E. Hermida A. Maltret A. Marchant J. Denjoy I. Champ-Rigot L. (2025). Clinical presentation and outcomes of patients with biallelic SCN5A variants: a systematic review *heart rhythm* . O2 6, 1372–1381. 10.1016/j.hroo.2025.06.010 PMC12635737 41280527

[B9] Uchino T. Zheng M. Q. Wang Y. Ono K. (2020). Cardiac specific transcription factor Csx/Nkx2.5 regulates transient-outward K+ channel expression in pluripotent P19 cell-derived cardiomyocytes. J. Physiol. Sci. 70 (1), 20. 10.1186/s12576-020-00748-z 32213161 PMC7096375

[B35] Uniat J. Hill A. Shwayder M. Bar-Cohen Y. (2024). Severe cardiac conduction disease associated with titin gene mutation. Pacing. Clin. Electrophysiol. 47 (2), 253–255. 10.1111/pace.14726 37221934

[B45] van den Boogaard M. Smemo S. Burnicka-Turek O. Arnolds D. E. van de Werken H. J. G. Klous P. (2014). A common genetic variant within SCN10A modulates cardiac SCN5A expression. J. Clin. Invest. 124, 1844–1852. 10.1172/JCI73140 24642470 PMC3973109

[B100] van Setten J. Brody J. A. Jamshidi Y. Swenson B. R. Butler A. M. Campbell H. (2018). PR interval genome-wide association meta-analysis identifies 50 loci associated with atrial and atrioventricular electrical activity. Nat. Commun. 9, 2904. 10.1038/s41467-018-04766-9 30046033 PMC6060178

[B107] Verdonschot J. A. J. Hazebroek M. R. Krapels I. P. C. Henkens M. T. H. M. Raafs A. Wang P. (2020). Implications of genetic testing in dilated cardiomyopathy *Circ.-GENOMIC precis. Med* . Circ. Genom. Precis. Med. 13, 476–487. 10.1161/CIRCGEN.120.003031 32880476

[B108] Villarreal-Molina T. García-Ordóñez G. P. Reyes-Quintero Á. E. Domínguez-Pérez M. Jacobo-Albavera L. Nava S. (2021). Clinical spectrum of SCN5A channelopathy in children with primary electrical disease and structurally normal hearts. Genes 13, 16. 10.3390/genes13010016 35052356 PMC8774384

[B109] Vincentz J. W. Firulli B. A. Toolan K. P. Arking D. E. Sotoodehnia N. Wan J. (2019). Variation in a left ventricle-specific Hand1 enhancer impairs GATA transcription factor binding and disrupts conduction system development and function. Circ. Res. 125, 575–589. 10.1161/CIRCRESAHA.119.315313 31366290 PMC6715539

[B25] Vincentz J. W. Clouthier D. E. Firulli A. B. (2020). Mis-Expression of a cranial neural crest cell-specific gene program in cardiac neural crest cells modulates hand factor expression, causing cardiac outflow tract phenotypes. J. Cardiovasc. Dev. Dis. 7 (2), 13. 10.3390/jcdd7020013 32325975 PMC7344951

[B110] Wang G. Q. Sun X. J. Zhong L. (2025). An Uncommon Atrioventricular Block Pattern Associated with a Novel Mutation in TTN. 10.1093/qjmed/hcae077 38608183

[B111] Walsh R. Mauleekoonphairoj J. Mengarelli I. Bosada F. M. Verkerk A. O. van Duijvenboden K. (2025). A rare noncoding enhancer variant in SCN5A contributes to the high prevalence of Brugada syndrome in Thailand. Circulation 151, 31–44. 10.1161/CIRCULATIONAHA.124.069041 39391988 PMC11670919

[B112] Wang L. Cai Y. Jian L. Cheung C. W. Zhang L. Xia Z. (2021). Impact of peroxisome proliferator-activated receptor-α on diabetic cardiomyopathy. Cardiovasc. Diabetol. 20, 2. 10.1186/s12933-020-01188-0 33397369 PMC7783984

[B113] Ward S. M. Fernando S. J. Hou T. Y. Duffield G. E. (2010). The transcriptional repressor ID2 can interact with the canonical clock components CLOCK and BMAL1 and mediate inhibitory effects on mPer1 expression. J. Biol. Chem. 285, 38987–39000. 10.1074/jbc.M110.175182 20861012 PMC2998095

[B24] Wright J. J. Kim J. Buchanan J. Boudina S. Sena S. Bakirtzi K. (2009). Mechanisms for increased myocardial fatty acid utilization following short-term high-fat feeding. Cardiovasc Res. 82 (2), 351–360. 10.1093/cvr/cvp017 19147655 PMC2675931

[B114] Yadin D. Guetta T. Petrover Z. Alcalai R. Seidman J. Seidman C. E. (2023). Effect of pharmacological heart failure drugs and gene therapy on Danon’s cardiomyopathy. Biochem. Pharmacol. 215, 115735. 10.1016/j.bcp.2023.115735 37572991

[B115] Yang P. Lou Y. Geng Z. Guo Z. Wu S. Li Y. (2024). Allele-specific suppression of variant MHC with high-precision RNA nuclease CRISPR-Cas13d prevents hypertrophic cardiomyopathy. Circulation 150, 283–298. 10.1161/CIRCULATIONAHA.123.067890 38752340 PMC11259241

[B116] Yuan L. Bu S. Du M. Wang Y. Ju C. Huang D. (2023). RNF207 exacerbates pathological cardiac hypertrophy *via* post-translational modification of TAB1. Cardiovasc. Res. 119, 183–194. 10.1093/cvr/cvac039 35352799

[B117] Zaytseva A. K. Kulichik O. E. Kostareva A. A. Zhorov B. S. (2024). Biophysical mechanisms of myocardium sodium channelopathies. Pflugers Arch. 476, 735–753. 10.1007/s00424-024-02930-3 38424322

[B118] Zhang J. Vincent K. P. Peter A. K. Klos M. Cheng H. Huang S. M. (2020). Cardiomyocyte expression of ZO-1 is essential for normal atrioventricular conduction but does not alter ventricular function. Circ. Res. 127, 284–297. 10.1161/CIRCRESAHA.119.315539 32345129 PMC7571547

[B119] Zhang L. Cheng X. Chen J. Zhou M. Qian T. Zhang Z. (2020). Left bundle pacing for left bundle branch block and intermittent third-degree atrioventricular block in a MYH7 mutation-related hypertrophic cardiomyopathy with restrictive phenotype in a child *front. Pediatr* . 8 10.3389/fped.2020.00312 PMC7308432 32612965

[B120] Zhang Y. Khan S. Liu Y. Siddique R. Zhang R. Yong V. W. (2021). Gap junctions and hemichannels composed of connexins and pannexins mediate the secondary brain injury following intracerebral hemorrhage biology 11 (27). 10.3390/biology11010027 PMC8772966 35053024

[B121] Zhu Y. (2022). Gap junction-dependent and -Independent functions of Connexin43 in biology biology. Biol. (Basel). 11, 283. 10.3390/biology11020283 PMC8869330 35205149

